# Gut Microbiota‐Derived Indole‐3‐Propionic Acid Alleviates Diabetic Osteoporosis Through Nrf2‐Mediated Ferroptosis Suppression

**DOI:** 10.1002/advs.77649

**Published:** 2026-09-08

**Authors:** Jinwu Bai, Qinyong You, Gao Si, Daole Hu, Ao Sun, Shilong Su, Ruideng Wang, Jixing Fan, Shan Gao, Tengjiao Zhu, Chun‐Li Song, Fang Zhou, Yang Lv

**Affiliations:** ^1^ Department of Orthopedics Peking University Third Hospital Beijing China; ^2^ Beijing Key Laboratory of Advanced Bioadaptable Orthopedic Implants Peking University Third Hospital Beijing China; ^3^ Department of Trauma Orthopedics Shenzhen Second People's Hospital (The First Affiliated Hospital of Shenzhen University) Shenzhen China

**Keywords:** diabetic osteoporosis, ferroptosis, gut microbiota, indole‐3‐propionic acid, Nrf2, oxidative stress, tryptophan metabolism

## Abstract

Diabetic osteoporosis combines impaired bone formation with disproportionate skeletal fragility, yet the microbial metabolites linking diabetes‐associated dysbiosis to bone dysfunction remain unclear. This study implicates indole‐3‐propionic acid (IPA), a gut microbiota‐derived tryptophan metabolite, in the maintenance of skeletal homeostasis under diabetic conditions. Circulating IPA was lower in diabetic mice and in a small exploratory cohort of patients with diabetic osteoporosis. IPA concentrations were positively associated with bone mass. Metagenomic profiling linked lower IPA to impaired microbial tryptophan metabolism and reduced Clostridium abundance. In diabetic mice, IPA supplementation improved trabecular microarchitecture and bone formation. In bone marrow mesenchymal stem cells subjected to high glucose and palmitate, IPA also restored GPX4 and SLC7A11 expression and was associated with recovery of Nrf2‐mediated antioxidant signaling. Pharmacological inhibition of Nrf2 substantially attenuated these anti‐ferroptosis and pro‐osteogenic effects. Together, these findings support a gut microbiota‐IPA‐Nrf2‐ferroptosis pathway linking altered microbial tryptophan metabolism to impaired osteogenesis. They provide a rationale for evaluating IPA as a potential therapeutic strategy for diabetic osteoporosis.

## Graphical abstract

Diabetes‐associated dysbiosis is accompanied by reduced *Clostridium* abundance, impaired microbial tryptophan metabolism, and lower circulating indole‐3‐propionic acid (IPA). Exogenous IPA improves trabecular microarchitecture and bone formation and restores Nrf2‐associated antioxidant signaling in bone marrow mesenchymal stem cells, limiting iron accumulation, lipid peroxidation, and ferroptosis while preserving osteogenic differentiation under diabetic stress.

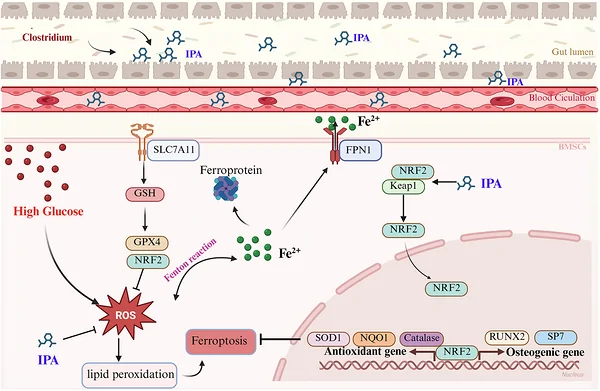

## 1 Introduction

Diabetes is increasingly acknowledged as a major contributor to skeletal fragility and fracture risk [1]. Patients with diabetes face a heightened risk of osteoporotic fractures, delayed fracture healing, and impaired bone regeneration [2]. Diabetes affects the skeleton by impairing trabecular architecture, tissue quality, remodeling activity, and regenerative potential, in addition to altering bone mineral density. These abnormalities may collectively account for the disproportionate skeletal fragility observed in diabetic individuals [3]. Type 2 diabetes can increase fracture risk even when areal bone mineral density is normal or relatively preserved, indicating that impaired bone quality, low‐turnover remodeling, and microarchitectural damage contribute substantially to skeletal fragility [1]. Chronic hyperglycemia, accumulation of advanced glycation end products, microvascular dysfunction, inflammation, and altered cellular metabolism have all been implicated in this phenotype [4]. Nevertheless, the biological mechanisms underlying diabetes‐associated bone deterioration remain incompletely understood, and effective strategies targeting the diabetic bone microenvironment are still limited [1].

Skeletal integrity is maintained through a dynamic balance between bone formation and resorption [5]. Bone marrow mesenchymal stromal cells (BMSCs) provide osteoblast‐lineage cells and are essential for bone development, remodeling, and repair [6]. Chronic hyperglycemia and lipid overload create a glucolipotoxic environment characterized by reactive oxygen species (ROS), mitochondrial dysfunction, inflammation, and metabolic disruption [7, 8]. Persistent redox imbalance can disrupt osteogenic transcription and matrix mineralization, potentially increasing susceptibility to ferroptosis [9, 10, 11], an iron‐dependent cell death process marked by phospholipid oxidation [12, 13, 14].

Nuclear factor erythroid 2‐related factor 2 (Nrf2) is a major transcriptional regulator of cellular redox homeostasis [15]. Activation of Nrf2 induces a broad antioxidant program involving glutathione metabolism, lipid peroxide detoxification, iron regulation, and other cytoprotective processes [16, 17]. Disruption of Nrf2 activity has been associated with defective osteogenesis and enhanced skeletal loss under diabetic conditions [18]. Reinforcement of Nrf2‐associated antioxidant defenses may therefore help BMSCs resist oxidative stress and ferroptosis [18]. However, the metabolic factors governing this response in diabetic bone remain poorly characterized.

The gut microbiome regulates both systemic metabolism and skeletal health [19, 20]. By releasing metabolites into the circulation, intestinal microorganisms communicate with distant tissues and influence immune, endocrine, metabolic, and redox processes [21, 22, 23]. Diabetes‐associated dysbiosis may therefore affect skeletal homeostasis not only through changes in microbial composition but also through alterations in metabolite production [24]. Beyond taxonomic dysbiosis, functional changes in microbial metabolite production may be particularly relevant to diabetic osteoporosis. Short‐chain fatty acids, secondary bile acids, and tryptophan‐derived indoles can influence intestinal barrier integrity, immune and endocrine signaling, cellular redox balance, and the activity of osteoblast‐ and osteoclast‐lineage cells [25]. Among these metabolites, microbial tryptophan‐derived indoles are of particular interest because of their reported antioxidant, anti‐inflammatory, and metabolic activities [26, 27, 28, 29]. Indole‐3‐propionic acid (IPA) is a tryptophan metabolite produced by certain anaerobic bacteria, including members of the genus Clostridium [30]. IPA protects against oxidative injury and improves metabolic homeostasis in obesity, insulin resistance, and diabetes [31, 32, 33]. Microbial tryptophan metabolism has also been linked to skeletal phenotypes [34, 35]. However, whether reduced IPA directly contributes to impaired osteogenesis and diabetic bone loss remains unknown. Because of its antioxidant properties, IPA may preserve osteogenesis by strengthening Nrf2‐mediated defenses and limiting ferroptosis. Whether IPA regulates ferroptosis in BMSCs, whether this effect requires Nrf2, and whether IPA is altered in diabetic osteoporosis remain unclear. We therefore combined clinical samples, diabetic mouse models, metagenomic and metabolite analyses, and BMSC experiments. We measured circulating IPA, tested whether supplementation improved bone microarchitecture and osteogenesis, and examined the roles of ferroptosis and Nrf2 signaling. Our findings identify a gut microbiota‐IPA‐Nrf2‐ferroptosis pathway linking microbial dysbiosis to impaired bone formation.

## 2 Results

### 2.1 Diabetes Is Associated With Gut Microbial Dysbiosis, Impaired Tryptophan Metabolism, and Low Circulating IPA

**FIGURE 1:**
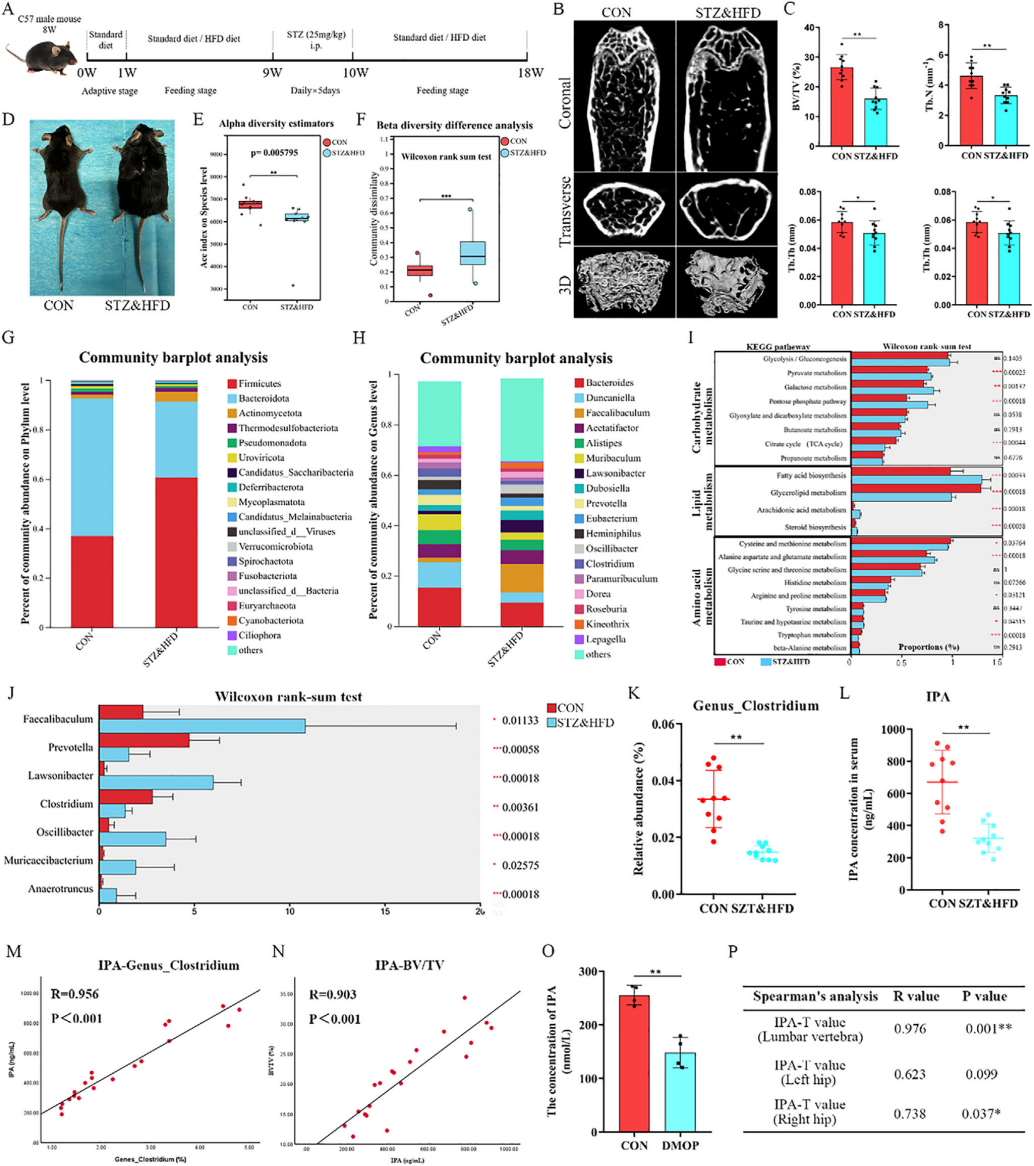
Gut microbial dysbiosis is associated with low circulating IPA and bone loss in diabetic osteoporosis. (A) Study design for generating diabetic osteoporosis in mice. (B) Distal‐femur micro‐CT images including sagittal views, coronal views, and 3D reconstruction views from CON and STZ&HFD mice (*n* = 10 per group). (C) Micro‐CT‐based quantification of trabecular bone parameters, including bone volume fraction (BV/TV), trabecular thickness (Tb. Th), trabecular number (Tb. N), and trabecular separation (Tb. Sp). (D) A general appearance photo of the mice at the end of the feeding stage. (E) α diversity index of the gut microbiota between two group. (F) β diversity of metagenomic profiles between two group. (G, H) Phylum‐ and genus‐level relative microbial abundance. (I) KEGG enrichment for differentially represented microbial pathways. (J) Wilcoxon rank‐sum comparisons at genus level. (K) Relative abundance of Clostridium. (L) Serum IPA concentrations. (M) Relationship between serum IPA and Clostridium abundance by Spearman analysis. (N) Spearman analysis of serum IPA vs. femoral BV/TV. (O) Serum IPA in healthy participants and patients with diabetic osteoporosis. (P) Spearman associations between IPA and bone mineral density at the specified sites. Values are mean ± SD. ^*^ *p* < 0.05; ^**^ *p* < 0.01.

A mouse model of type 2 diabetes‐associated osteoporosis was established using the high‐fat diet and STZ protocol depicted in Figure 1A. STZ&HFD mice developed a characteristic diabetic phenotype, including pronounced obesity (Figure 1D) and significantly greater body weight than CON mice (Figure S1A). Consistent with successful diabetes induction, fasting blood glucose exceeded 7.0 mmol/L, random blood glucose exceeded 11.1 mmol/L, and oral glucose tolerance was markedly impaired in STZ&HFD mice compared with controls (Figure S1B–D). These findings confirmed the diabetic metabolic phenotype. We next assessed the skeletal phenotype of diabetic mice using micro‐computed tomography. Micro‐CT revealed significant trabecular bone loss in the distal femur (Figure 1B). The trabecular microarchitecture showed signs of deterioration, as evidenced by decreased bone volume/total volume (BV/TV), trabecular thickness (Tb. Th), and trabecular number (Tb. N), together with an increase in trabecular separation (Tb. Sp) (Figure 1C).

Given the emerging role of the gut‐bone axis in skeletal homeostasis, we next characterized changes in the gut microbiota using whole‐metagenome sequencing. α‐diversity and β‐diversity differed significantly between control and diabetic mice (Figure 1E,F). Multiple ordination methods consistently distinguished the two microbial communities (Figure S2A–C). Consistent with these observations, diabetic mice had a significantly lower microbial health index and a higher dysbiosis index, indicating extensive disruption of gut microbial homeostasis (Figure S2D,E). Taxonomic analysis identified broad compositional shifts. At the phylum level, the Firmicutes‐to‐Bacteroidetes ratio was significantly lower in STZ&HFD mice (Figures 1G and S2F). More importantly, several bacterial genera involved in microbial metabolism were also markedly altered. The relative abundances of Bacteroides, Prevotella, and Clostridium were significantly lower, whereas Faecalibaculum and Lawsonibacter were enriched in diabetic mice (Figure 1H,J,K). Functional annotation identified changes in carbohydrate, lipid, and amino acid metabolism, including reduced tryptophan metabolism (Figure 1I).

Given the growing recognition of microbial tryptophan metabolites in metabolic homeostasis and the capacity of certain Clostridium species to produce IPA, we investigated whether circulating IPA was altered in diabetic mice. Serum IPA concentrations were markedly lower in STZ&HFD mice than in controls (Figure 1L). Spearman correlation analysis showed that circulating IPA concentrations were positively correlated with the relative abundance of Clostridium (Figure 1M). Notably, serum IPA was also positively associated with BV/TV (Figure 1N), linking low circulating IPA to impaired skeletal integrity under diabetic conditions. To assess the clinical relevance of these findings, serum IPA concentrations were measured in healthy individuals and patients with diabetic osteoporosis. Participant demographic and clinical data are presented in Table 1. Consistent with the findings in mice, circulating IPA concentrations were significantly lower in patients with diabetic osteoporosis (Figure 1O). Serum IPA concentrations were positively correlated with bone mineral density at the lumbar spine and right hip. A similar trend was observed at the left hip but did not reach statistical significance (Figure 1P). These exploratory data associate diabetic osteoporosis with impaired microbial tryptophan metabolism and low circulating IPA.

**TABLE 1:** Baseline characteristics of participants in the cross‐sectional study.

Item	CON (*n* = 4)	DMOP (*n* = 4)
Age	63.5 ± 6.61	67.75 ± 7.89
Male	1(25%)	1(25%)
Female	3(75%)	3(75%)
BMI (kg/m^2^)	28.12 ± 2.65	24.98 ± 2.18
HbA1c (%)	6.07 ± 0.15	7.8 ± 0.54^**^
Fasting blood glucose (mmol/L)	5.5 ± 0.73	11.59 ± 3.11^**^
Systolic blood pressure (mmHg)	136.75 ± 3.2	136.25 ± 17.5
Diastolic blood pressure (mmHg)	81 ± 5.66	80.75 ± 5.25
Lumbar spine T‐score	−0.48 ± 1.18	−2.85 ± 0.6^*^
Left hip T‐score	−1.08 ± 0.36	−2.05 ± 0.79
Right hip T‐score	−1.05 ± 0.31	−2.23 ± 0.76^*^

Abbreviations: CON, healthy control group; DMOP, type 2 diabetes mellitus with osteoporosis.

*Note*: Data are mean ± SD. ^*^ *p* < 0.05 and ^**^ *p* < 0.01 vs. CON.

### 2.2 Diabetic Bone Loss Is Accompanied by Impaired Osteogenesis and a Ferroptosis‐associated Phenotype

**FIGURE 2:**
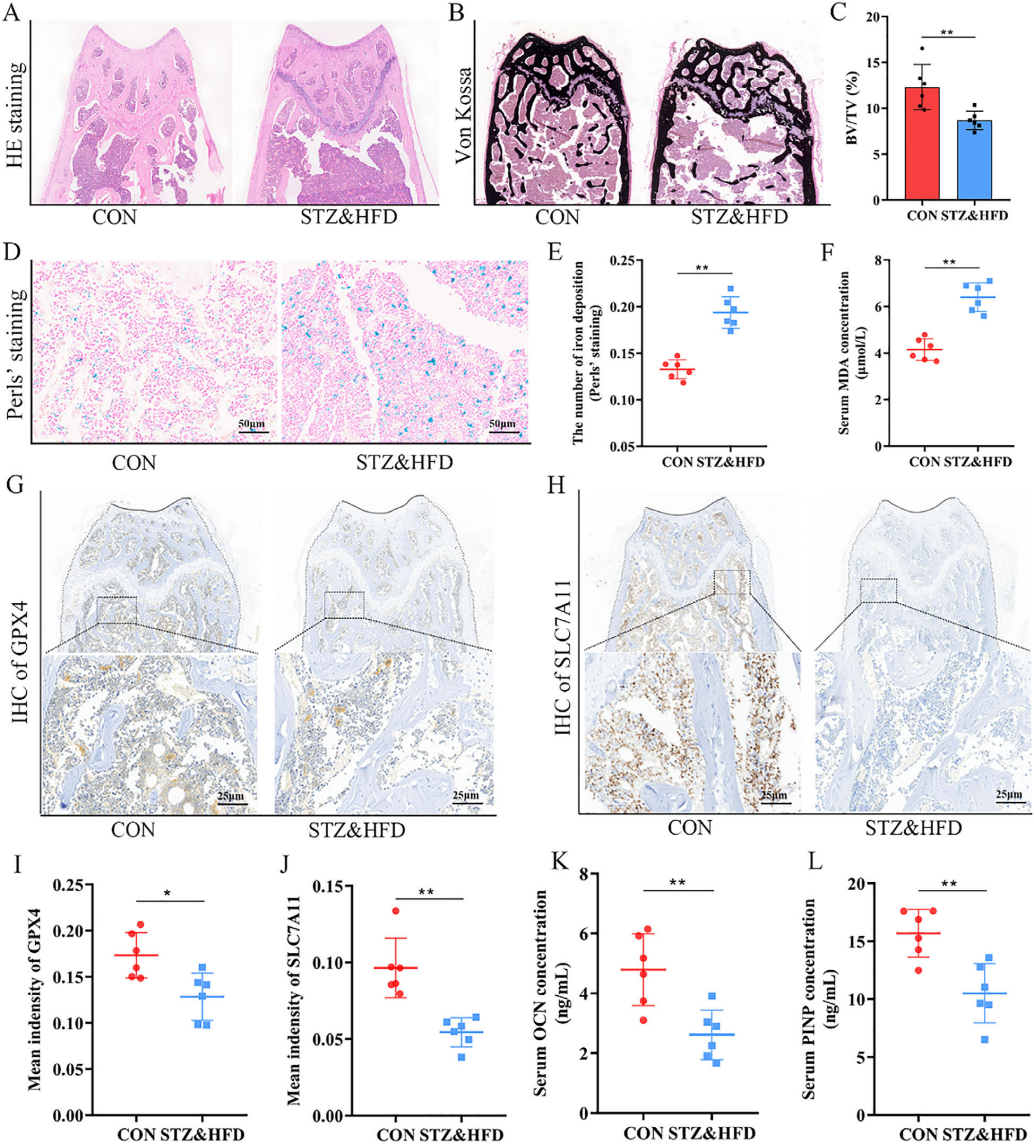
Diabetic osteoporosis is accompanied by impaired osteogenesis and a ferroptosis‐associated skeletal phenotype. (A) H&E‐stained distal femora from CON and STZ&HFD mice. (B) Representative von Kossa staining of mineralized bone. (C) Histomorphometric BV/TV derived from von Kossa sections. (D) Prussian blue detection of iron deposits (scale bar, 50 µm). (E) Prussian blue‐positive area. (F) Serum MDA between two group. (G,H) GPX4 and SLC7A11 immunohistochemistry in the distal femoral metaphysis (scale bar, 25 µm). (I,J) Quantified GPX4‐ and SLC7A11‐positive staining. (K,L) Circulating OCN and PINP. Values are mean ± SD. ^*^ *p* < 0.05; ^**^ *p* < 0.01.

To further characterize the pathological changes associated with diabetes‐related bone loss, we performed histological analyses of the distal femur. H&E staining revealed markedly reduced trabecular bone in STZ&HFD mice compared with CON mice (Figure 2A). Consistently, von Kossa staining showed less mineralized bone within the trabecular compartment of diabetic mice (Figure 2B), Quantitative analysis confirmed a significant reduction in BV/TV (Figure 2C). These histological findings corroborated the micro‐CT results. Circulating OCN and PINP concentrations were also lower, consistent with reduced bone formation (Figure 2K,L). Given the association between oxidative stress and ferroptosis in diabetes, we next examined ferroptosis‐associated changes in diabetic bone. Prussian blue staining revealed greater iron deposition in the distal femur of STZ&HFD mice than in CON mice (Figure 2D,E). Serum malondialdehyde (MDA) concentrations were also markedly higher in diabetic mice (Figure 2F), indicating increased lipid peroxidation. We further evaluated GPX4 and SLC7A11 expression by immunohistochemical staining (Figure 2G,H). Both proteins were markedly less abundant in the trabecular bone of STZ&HFD mice than in controls (Figure 2I,J), indicating impaired defense against lipid peroxidation. Together, these findings associate diabetic skeletal deterioration with iron accumulation, lipid peroxidation, and impaired ferroptosis defense.

### 2.3 IPA Restores Osteogenic Differentiation Under Diabetic Stress

**FIGURE 3:**
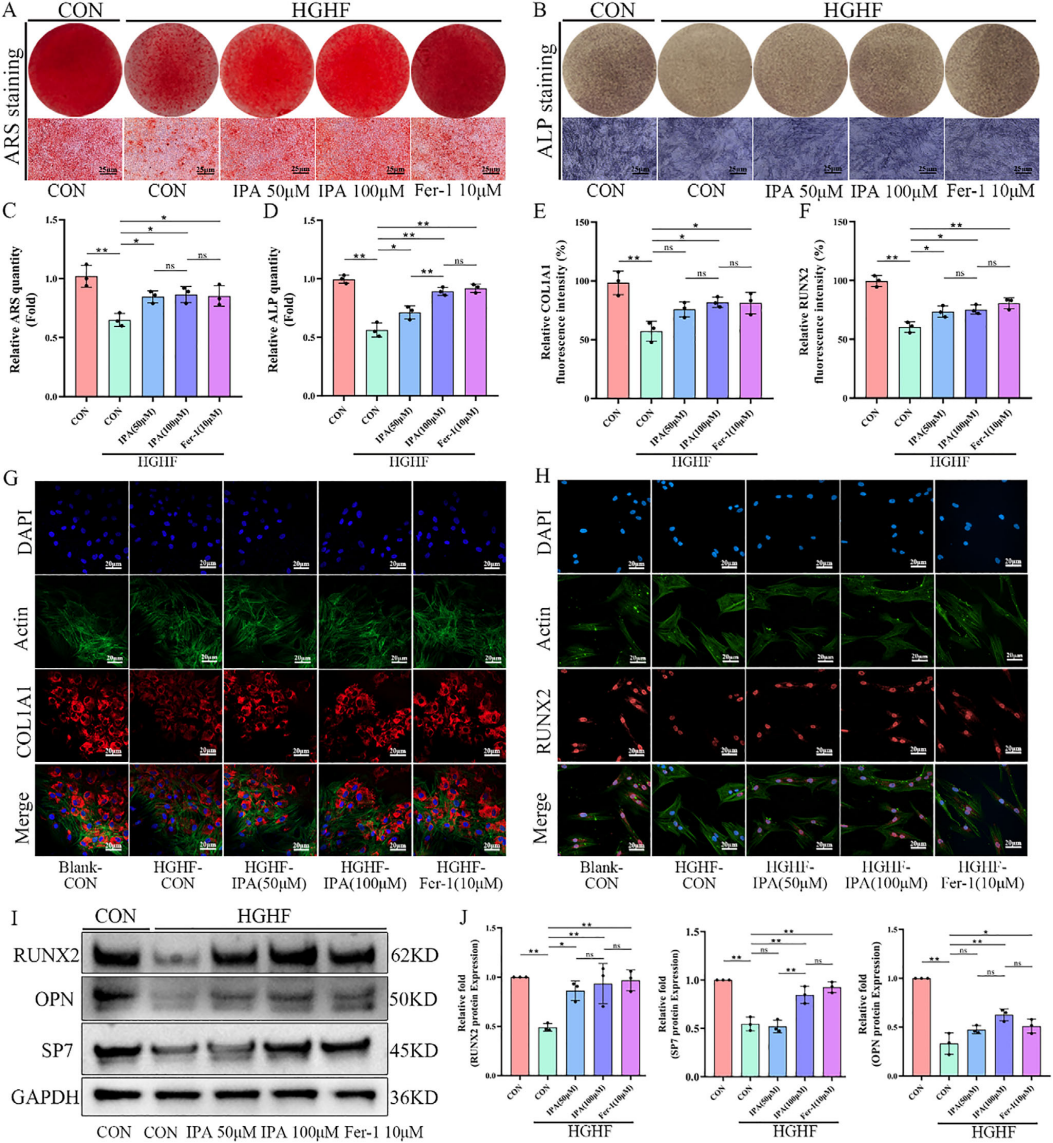
IPA restores BMSC osteogenic differentiation under diabetic stress. (A) ARS staining following osteogenic induction (scale bar, 25 µm). (B) ALP staining (scale bar, 25 µm). (C,D) Quantification of ARS and ALP activity. (E,F) Quantification of RUNX2‐ and COL1A1‐positive fluorescence intensity. (G,H) Representative immunofluorescence staining of RUNX2 and COL1A1 in BMSCs. Nuclei were counterstained with DAPI. Scale bar, 20 µm. (I) Western blot analysis of RUNX2, OPN, and SP7 after IPA or Fer‐1 exposure under CON or HGHF conditions. (J) Based on the quantitative analysis of protein bands by Western blot, normalized to GAPDH. Values are mean ± SD. All experiments were repeated at least three times. ^*^ *p* < 0.05; ^**^ *p* < 0.01.

To determine whether IPA protects against diabetes‐associated osteogenic dysfunction, we exposed BMSCs to HGHF to model the diabetic microenvironment in vitro. Before the functional experiments, the cytotoxicity of IPA and the ferroptosis inhibitor ferrostatin‐1 (Fer‐1) was assessed using the CCK‐8 assay. IPA showed no detectable cytotoxicity at concentrations up to 100 µmol/L (Figure S3A). By contrast, Fer‐1 significantly reduced cell viability at 20 µmol/L, whereas 10 µmol/L had no detectable cytotoxic effect and was therefore used in subsequent experiments (Figure S3B). ARS staining showed that HGHF exposure markedly impaired extracellular matrix mineralization in BMSCs (Figure 3A). Notably, IPA treatment substantially restored mineralized nodule formation in a concentration‐dependent manner (Figure 3C). Fer‐1 produced a comparable improvement. Consistently, ALP staining and quantitative analysis showed that both IPA and Fer‐1 restored the early osteogenic activity suppressed by HGHF (Figure 3B,D).

Immunofluorescence staining revealed that HGHF markedly suppressed the expression of RUNX2 and COL1A1, whereas treatment with either IPA or Fer‐1 effectively restored the expression of both proteins (Figure 3E–H). Western blot analysis further confirmed these observations, showing that HGHF‐induced downregulation of osteogenic proteins was significantly reversed following IPA treatment (Figure 3I,J). Notably, the protective effect of 100 µM IPA was comparable to that achieved with Fer‐1. The transcriptional changes were consistent with these protein‐level observations. Quantitative PCR demonstrated that the osteogenic genes Runx2, Ocn, and Sp7 were significantly downregulated following HGHF stimulation, whereas both IPA and Fer‐1 markedly restored their expression (Figure S4A–C). These findings show that IPA preserves BMSC osteogenic differentiation under glucolipotoxic conditions. The response to Fer‐1 further implicates ferroptosis in this effect.

### 2.4 IPA Suppresses Ferroptosis in BMSCs Exposed to Diabetic Stress

**FIGURE 4:**
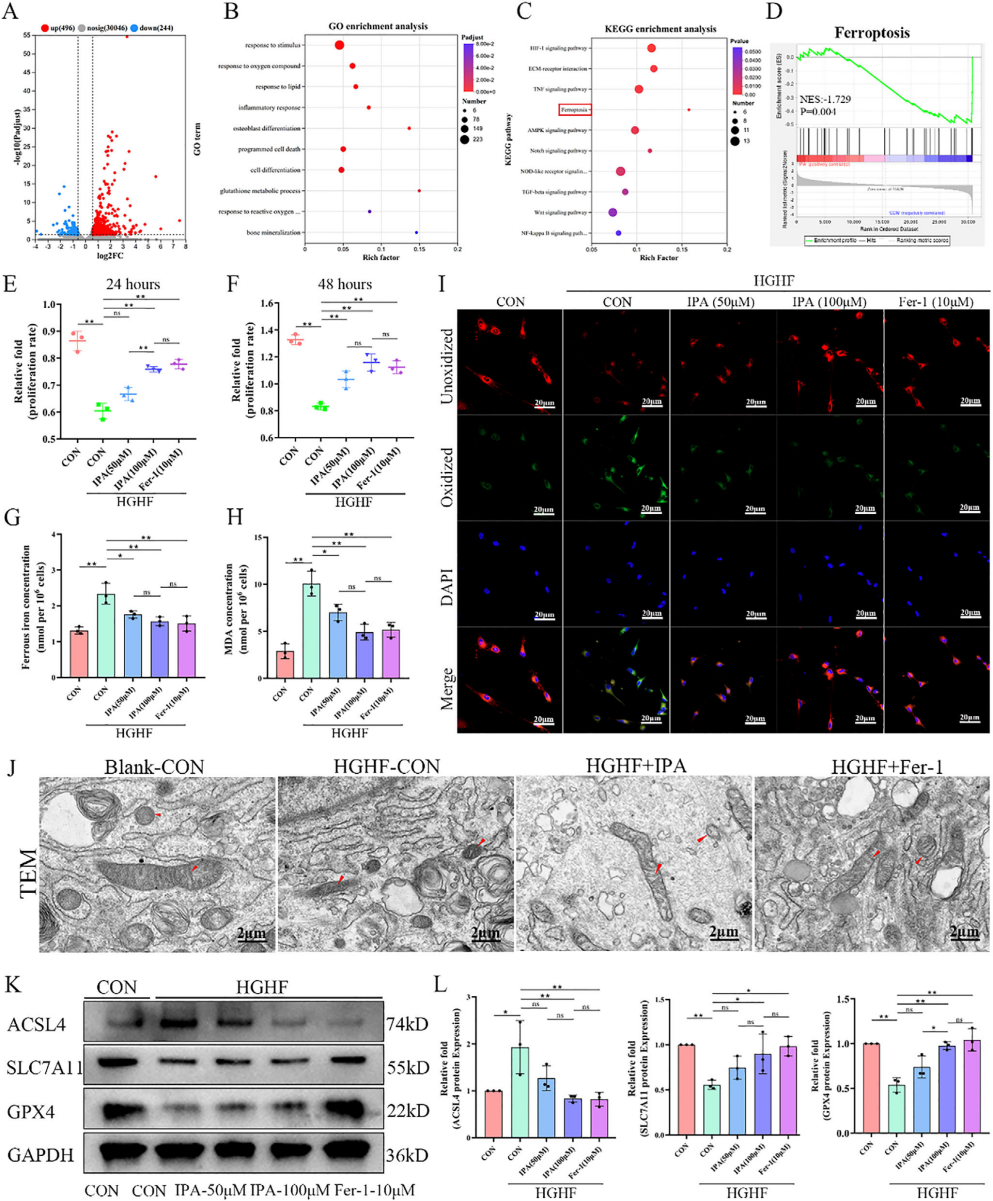
IPA suppresses ferroptosis in BMSCs exposed to diabetic stress. (A) Volcano plot of genes differentially expressed after IPA treatment under HGHF conditions. (B) Gene Ontology enrichment analysis. (C) KEGG pathway enrichment analysis. (D) GSEA analysis of the ferroptosis pathway. (E,F) Cell viability was assessed at 24 and 48 h using the CCK8 assay. (G) Intracellular Fe2^+^ level of BMSCs. (H) Intracellular MDA concentration of BMSCs. (I) Lipid‐peroxidation images of BMSCs (scale bar, 20 µm). (J) Transmission electron microscopy of mitochondrial ultrastructure. Scale bar, 2.0 µm. (K) Western blots of GPX4, SLC7A11 and ACSL4 protein. (L) Based on the quantitative analysis of protein bands by Western blot, normalized to GAPDH. All experiments were repeated at least three times. ^*^ *p* < 0.05; ^**^ *p* < 0.01.

We conducted RNA sequencing to explore the mechanisms through which IPA exerts its effects. Differential expression analysis identified broad transcriptional changes after IPA treatment (Figures 4A and S5A). Gene Ontology terms included regulated cell death, osteogenic differentiation, oxidative stress, and inflammation. KEGG analysis revealed enrichment in pathways such as ferroptosis, TNF signaling, and NF‐κB signaling (Figure 4B,C). Compared with HGHF alone, IPA increased the expression of genes involved in antioxidant defense, glutathione synthesis, and iron homeostasis. These genes included Hmox1, Slc40a1, Slc7a11, Gclm, and Gclc (Figure S5B). Gene set enrichment analysis indicated reduced enrichment of the ferroptosis gene set following IPA treatment (Figure 4D).

Functional assays validated the transcriptomic findings. HGHF decreased BMSC viability, while IPA increased viability in a concentration‐dependent manner. Fer‐1 produced a similar protective effect (Figure 4E,F). HGHF also increased intracellular Fe^2^^+^ and MDA (Figure 4G,H). IPA and Fer‐1 each reduced iron accumulation and lipid peroxidation. Lipid ROS likewise increased after HGHF exposure and decreased after either treatment (Figure 4I). Transmission electron microscopy (TEM) revealed that HGHF‐treated BMSCs had shrunken mitochondria, increased membrane density, and loss of cristae (Figure 4J). IPA and Fer‐1 largely reversed these ferroptosis‐associated ultrastructural changes and preserved mitochondrial integrity.

At the protein level, HGHF reduced GPX4 and SLC7A11 and increased ACSL4, whereas IPA reversed these changes (Figure 4K,L). Immunofluorescence showed that IPA restored GPX4 and FTH1 expression (Figure S6A,B), and RT‐qPCR confirmed corresponding changes in ferroptosis‐related transcripts (Figure S7). Together, these data show that IPA limits iron accumulation, lipid peroxidation, mitochondrial injury, and ferroptosis‐associated molecular changes in BMSCs under diabetic stress.

### 2.5 IPA Restores Nrf2 Signaling to Suppress Ferroptosis Under Diabetic Stress

**FIGURE 5:**
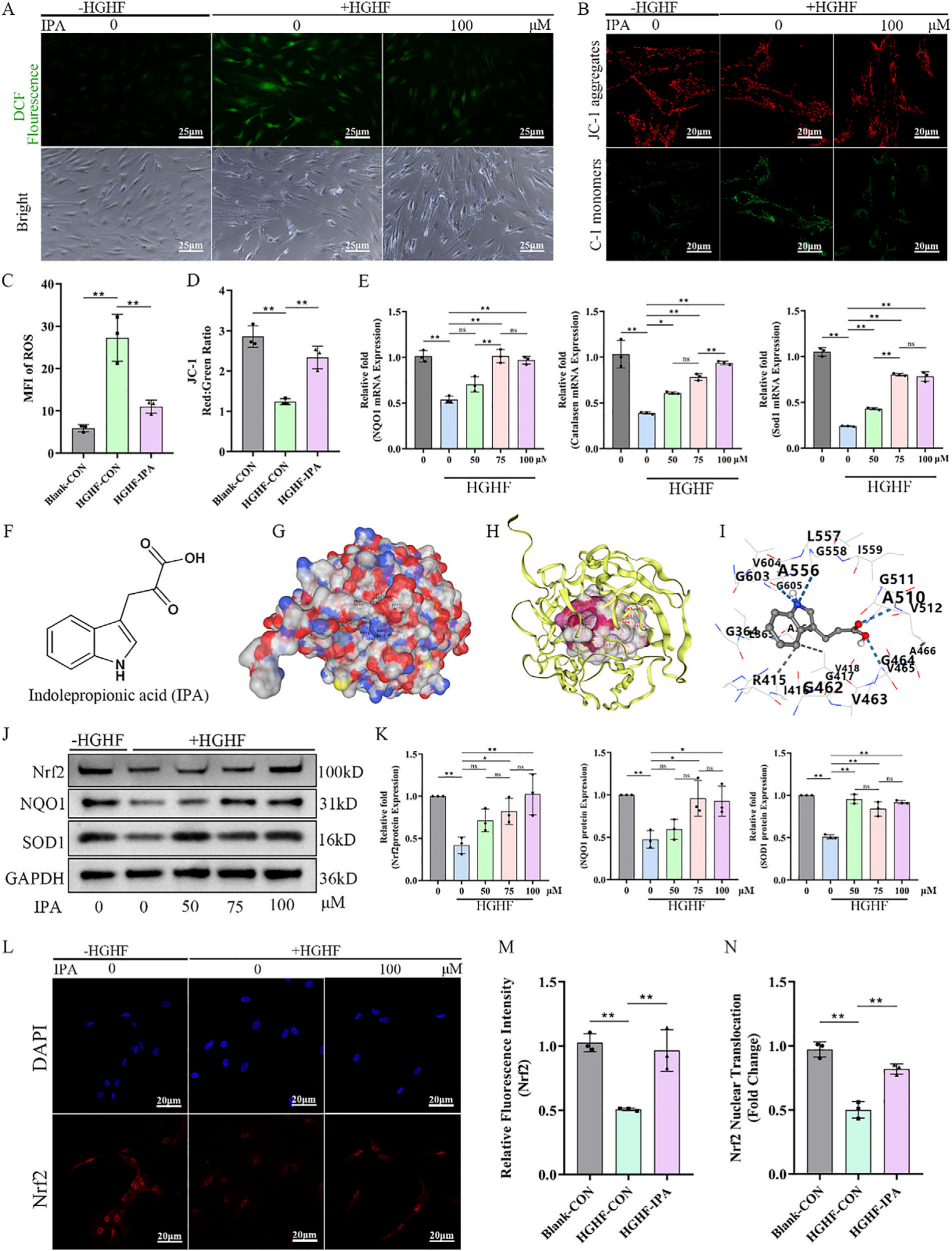
IPA activates Nrf2 signaling and suppresses ferroptosis under diabetic stress. (A) Representative DCFH‐DA images of intracellular ROS. Scale bar, 25 µm. (B) Representative JC‐1 images of mitochondrial membrane potential. Scale bar, 20 µm. (C) Quantification of ROS intensity in BMSCs. (D) Quantification of Mitochondrial membrane potential based on the JC‐1 fluorescence. (E) RT‐qPCR analysis of Nqo1, Catalase, and Sod1. (F) Chemical structure of IPA. (G) Three‐dimensional model of IPA docked to the KEAP1 Kelch‐Neh2 complex. (H,I) Predicted binding pose and hydrogen bonds with Val465, Val512, Leu557 and Val604. (J) Western blots of Nrf2, NQO1 and SOD1 protein. (K) Based on the quantitative analysis of protein bands by Western blot, normalized to GAPDH. (L) Representative Nrf2 immunofluorescence images. Nuclei were counterstained with DAPI. Scale bar, 20 µm. (M,N) Quantification of Nrf2 fluorescence and nuclear localization. All experiments were repeated at least three times. ^*^ *p* < 0.05; ^**^ *p* < 0.01.

Because IPA strongly altered oxidative‐stress pathways, we examined cellular redox homeostasis. HGHF elevated intracellular ROS levels, while IPA decreased ROS in a dose‐dependent manner (Figure 5A,C). IPA also partially restored the mitochondrial membrane potential reduced by HGHF (Figure 5B,D). HGHF reduced the expression of Nqo1, Catalase, and Sod1 transcripts, while IPA reinstated their levels in a concentration‐dependent manner (Figure 5E). Molecular docking was used to examine the potential interaction between IPA and the KEAP1 Kelch domain. IPA had a predicted binding energy of −7.0 kcal/mol and formed predicted hydrogen bonds with Val465, Val512, Leu557, and Val604 (Figure 5F–I). These computational data suggest a possible interaction but do not establish direct binding.

Western blotting showed that HGHF reduced Nrf2, NQO1, and SOD1, whereas IPA restored their abundance in a concentration‐dependent manner (Figure 5J,K). Immunofluorescence further showed that IPA restored Nrf2 abundance and nuclear localization after HGHF exposure (Figure 5L–N). We used ML385 to test the functional contribution of Nrf2. ML385 inhibited Nrf2 at 5–10 µmol/L, but 10 µmol/L reduced cell viability. Subsequent experiments therefore used 5 µmol/L (Figures S8A,B and S9). Nrf2 inhibition substantially weakened the IPA‐mediated recovery of mineralization and ALP activity (Figure S10A–D). ML385 also reversed the effects of IPA on GPX4, SLC7A11, and ACSL4 (Figure S11A,B). These data support an important contribution of Nrf2 to IPA‐mediated ferroptosis suppression and osteogenic maintenance.

### 2.6 IPA Attenuates Diabetic Bone Loss and Improves Bone Formation in Vivo

**FIGURE 6:**
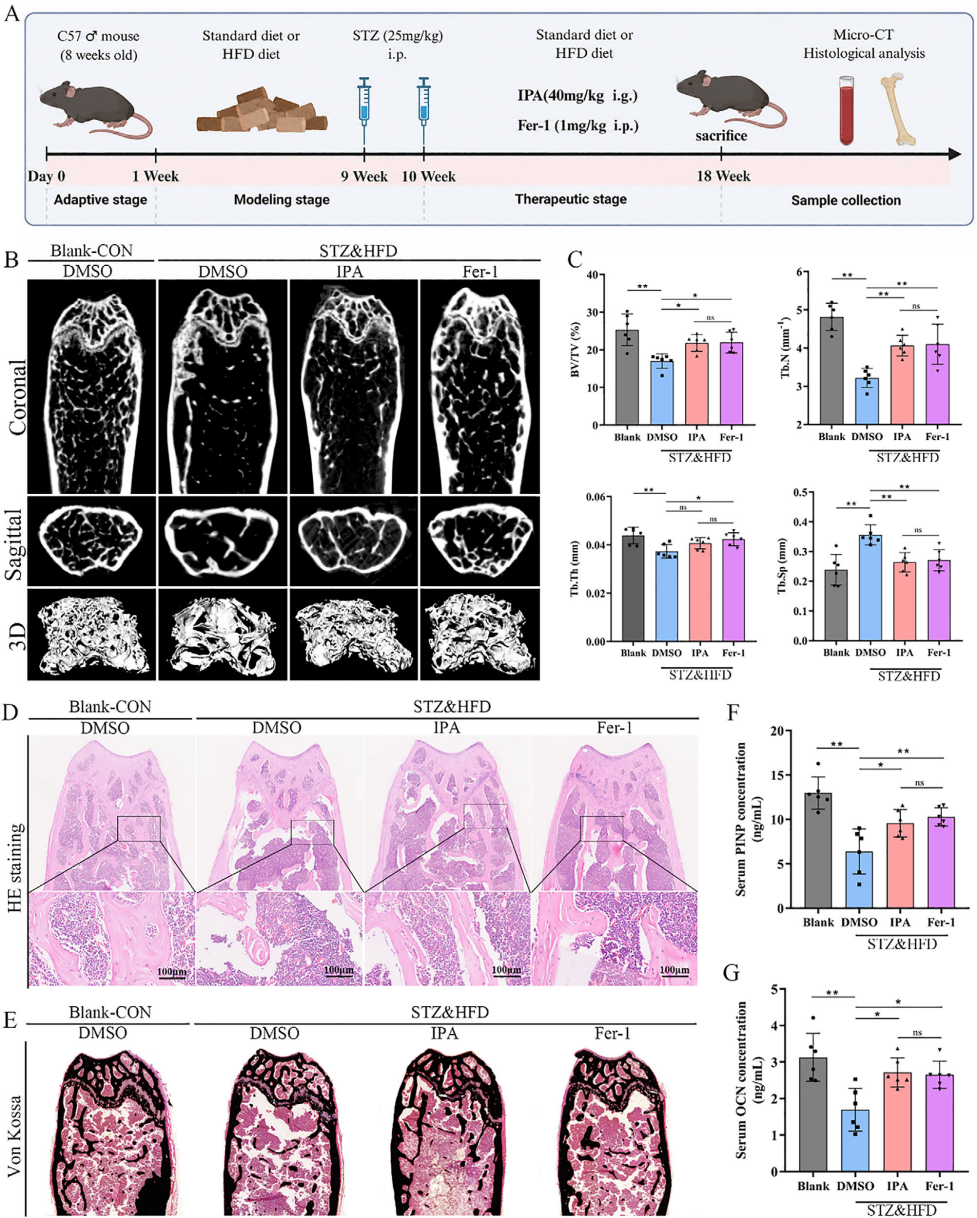
IPA attenuates diabetic bone loss and improves bone formation in vivo. (A) The flowchart of the entire animal intervention experiment (B) Distal‐femur micro‐CT images including sagittal views, coronal views and 3D reconstruction views from CON, STZ&HFD, STZ&HFD+IPA, and STZ&HFD+Fer‐1 mice (*n* = 6 per group). (C) Quantification of bone trabeculae including BV/TV, Tb. Th, Tb. N and Tb. Sp from four groups. (D) Representative HE staining of distal femur. Scale bar, 100 µm. (E) Representative Von Kossa staining images of distal femur. (F,G) Serum PINP and OCN concentrations of four groups. Data are mean ± SD. ^*^ *p* < 0.05; ^**^ *p* < 0.01.

To validate the osteoprotective effects of IPA in vivo, we generated a second cohort of diabetic mice with the same high‐fat diet and STZ protocol. Mice then received IPA or Fer‐1 (Figure 6A). Diabetic mice remained heavier and hyperglycemic (Figure S12A–C). IPA modestly reduced body weight and blood glucose, but neither difference was statistically significant. Its skeletal effects were therefore unlikely to reflect improved glycemic control alone. Micro‐CT showed severe trabecular deterioration in untreated diabetic mice. Both IPA and Fer‐1 increased BV/TV, Tb.Th, and Tb.N, while decreasing Tb.Sp (Figure 6B,C). No statistically significant differences were observed between the two intervention groups.

Histological evaluation corroborated the imaging observations. Diabetic mice exhibited lower trabecular bone volume on H&E sections and reduced matrix mineralization by von Kossa staining. IPA and Fer‐1 improved both outcomes (Figures 6D,E and S13B). Serum PINP and OCN were lower in diabetic mice and increased after either intervention (Figure 6F,G). Conversely, the elevated CTX‐I level decreased after IPA or Fer‐1 treatment (Figure S13A). These findings show that IPA protects against diabetes‐associated skeletal deterioration and improves bone remodeling in vivo. The broadly similar response to Fer‐1 is consistent with a role for ferroptosis in diabetic bone loss.

### 2.7 IPA Preserves Osteogenic Capacity and Reduces Skeletal Ferroptosis in Diabetic Mice

**FIGURE 7:**
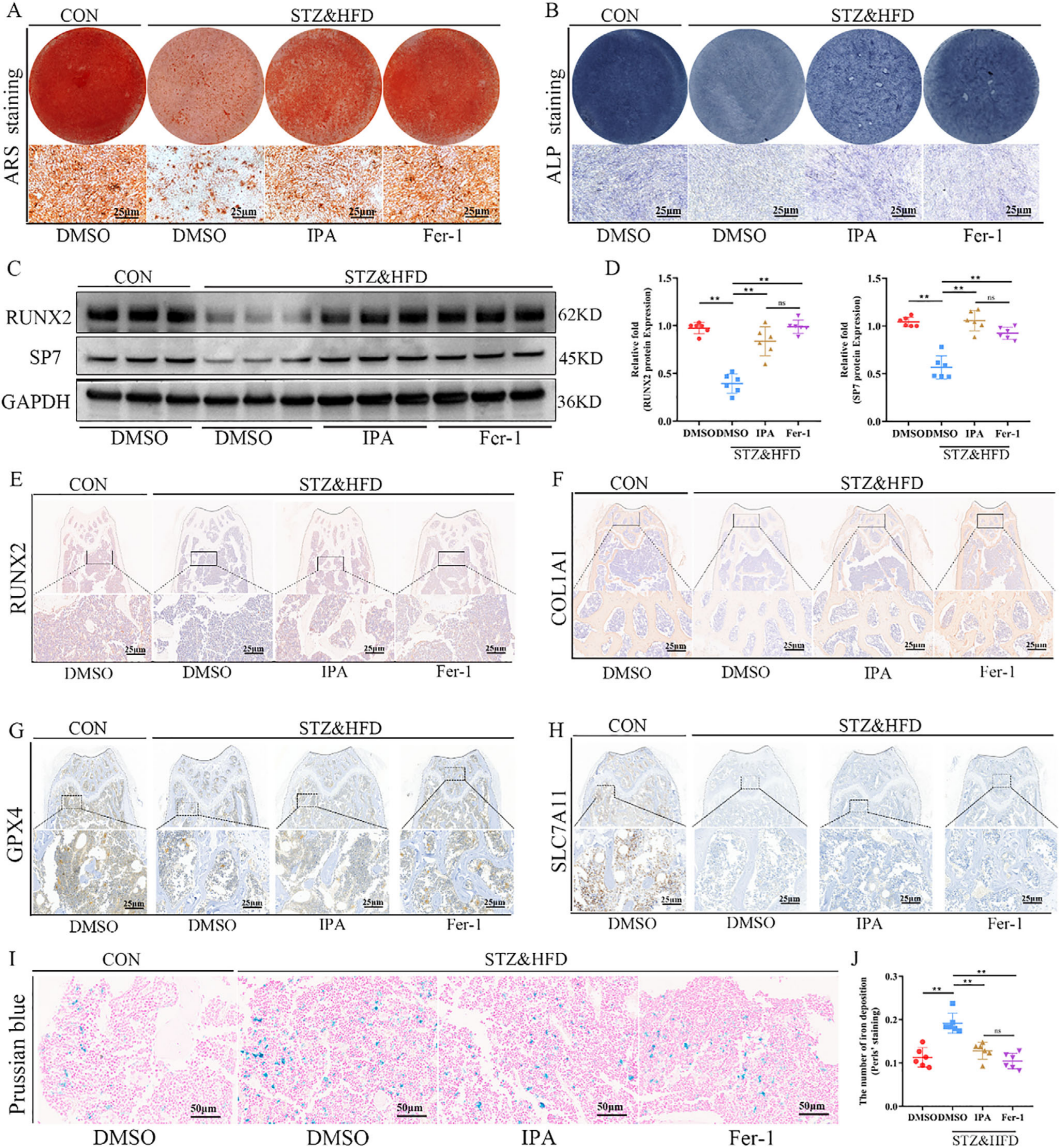
IPA preserves osteogenic potential and reduces skeletal ferroptosis in diabetic mice. (A) Representative ARS staining images of BMSCs isolated from CON, STZ&HFD, STZ&HFD+IPA, and STZ&HFD+Fer‐1 mice after osteogenic induction. Scale bar, 25 µm. (B) Representative ALP staining images of BMSCs isolated from the four groups. Scale bar, 25 µm. (C) The expression of RUNX2 and SP7 protein of BMSCs isolated from four groups by Western blot. (D) Based on the quantitative analysis of protein bands by Western blot, normalized to GAPDH. (E,F) Representative immunohistochemical staining of RUNX2 and COL1A1 in distal femoral metaphyses. Scale bar, 25 µm. (G,H) Representative immunohistochemical staining of GPX4 and SLC7A11 in distal femoral metaphyses. Scale bar, 25 µm. (I) Representative Prussian blue staining of femoral iron deposition. Scale bar, 50 µm. (J) Quantification of Prussian blue‐positive area. Data are mean ± SD. ^*^ *p* < 0.05; ^**^ *p* < 0.01.

To determine whether systemic IPA preserved the intrinsic osteogenic capacity of BMSCs, we isolated primary cells from each group and differentiated them ex vivo. BMSCs from diabetic mice showed reduced mineralized nodule formation and ALP activity. Cells from IPA‐ or Fer‐1‐treated mice showed greater osteogenic differentiation (Figures 7A,B and S14A,B). RUNX2 and SP7 expression was also lower in BMSCs from diabetic mice and restored after either treatment (Figure 7C,D). Immunohistochemistry of distal femora showed corresponding recovery of RUNX2 and COL1A1 (Figure 7E,F).

We next examined ferroptosis‐associated changes in bone. GPX4 and SLC7A11 were lower in diabetic femora and restored by IPA or Fer‐1 (Figure 7G,H). Both treatments also reduced diabetes‐associated iron deposition in trabecular bone (Figure 7I,J). Histological analysis showed no detectable treatment‐related lesions in the heart, liver, spleen, lungs, or kidneys (Figure S15). Within the limits of this assessment, neither intervention produced detectable histological toxicity. These findings show that IPA preserves BMSC osteogenic potential and reduces ferroptosis‐associated skeletal injury in diabetic mice.

## 3 Discussion

**FIGURE 8:**
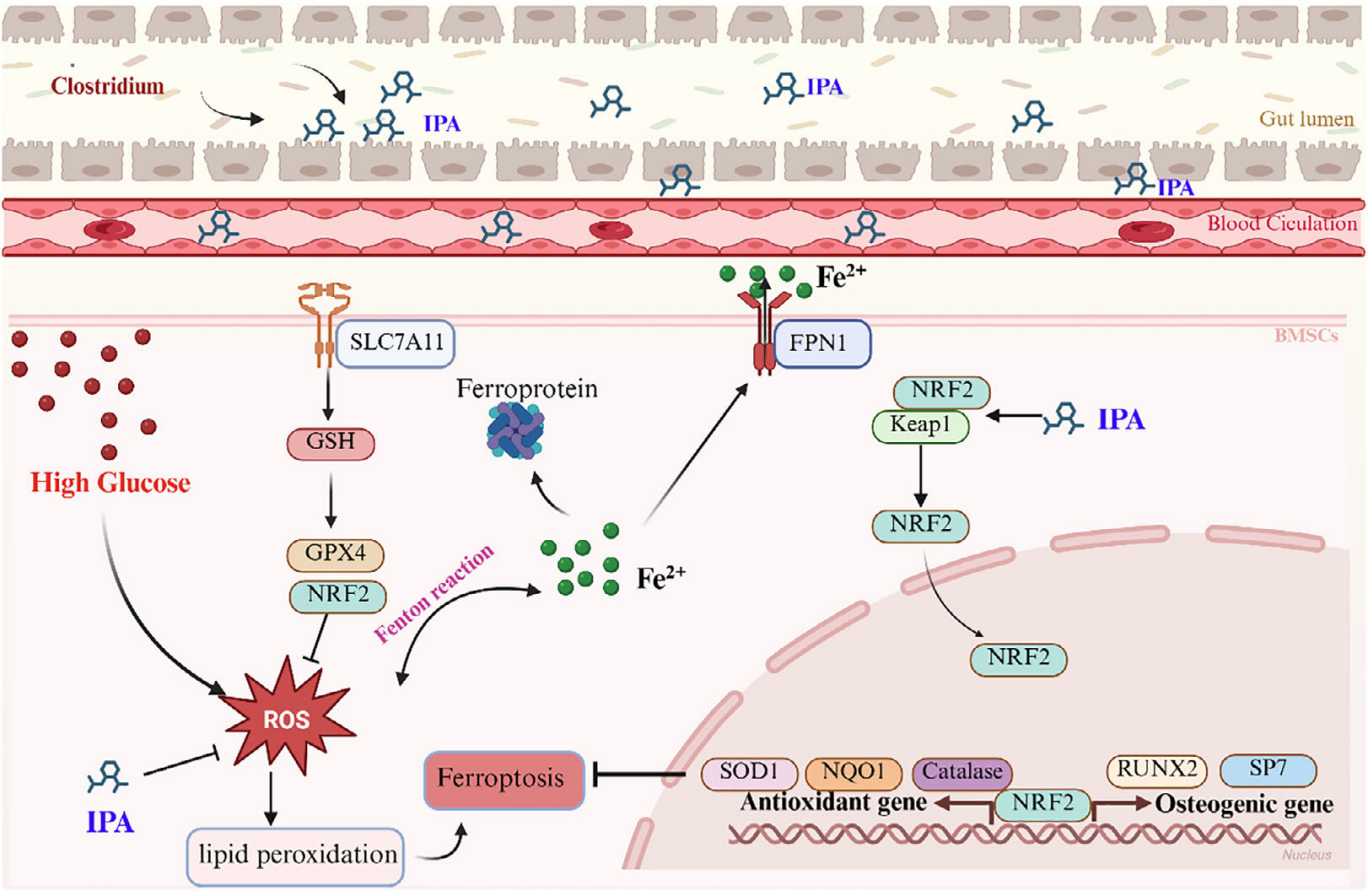
Schematic illustration of gut microbiota‐IPA‐Nrf2‐ferroptosis pathway linking diabetic dysbiosis to impaired osteogenesis.

This study identifies reduced availability of the microbial tryptophan metabolite IPA as a metabolic feature of diabetic osteoporosis. Diabetes was accompanied by gut dysbiosis, reduced Clostridium abundance, impaired microbial tryptophan metabolism, and lower circulating IPA. These changes were associated with poorer trabecular microarchitecture in mice and lower bone mineral density in an exploratory clinical cohort. IPA supplementation improved bone microarchitecture and preserved BMSC osteogenic capacity under diabetic conditions. These effects were associated with restored Nrf2‐mediated antioxidant defense and reduced ferroptosis (Figure 8). IPA did not significantly affect body weight or blood glucose levels, suggesting that its skeletal benefits were not solely due to systemic metabolic improvements.

The gut microbiota regulates host metabolism and contributes to metabolic bone disease [36]. In diabetes, gut microbial disruption accompanies chronic inflammation, metabolic imbalance, and impaired tissue repair [37], processes linked to skeletal deterioration [38]. Growing evidence suggests that altered microbial metabolism may be as important as compositional change. Host‐microbe communication is mediated by short‐chain fatty acids, secondary bile acids, and tryptophan metabolites [39], which also impact bone remodeling via immune, metabolic, and redox pathways [40]. Tryptophan‐derived indoles are especially relevant because they enter the circulation and modulate barrier integrity, inflammation, oxidative stress, and metabolism [41]. Gut bacteria metabolize dietary tryptophan into indole‐3‐acetic acid, indole‐3‐lactic acid, indoleacrylic acid, and IPA [42, 43]. Reduced circulating IPA has been reported in diabetes [33], cardiovascular disease [32, 44, 45], and neurodegenerative disorders [46, 47]. Previous skeletal studies have focused mainly on osteoclast formation and bone resorption [22, 35, 48]. Our findings extend this work by associating IPA with preserved osteoblast‐lineage function and BMSC osteogenesis under glucolipotoxic stress.

The parallel reductions in Clostridium abundance, microbial tryptophan metabolism, and circulating IPA suggest that diabetic dysbiosis may reduce IPA availability. However, our data do not show that loss of a specific Clostridium strain caused the reduction. Clostridium is a heterogeneous genus, and IPA production is strain‐dependent [49]. Circulating IPA also depends on dietary tryptophan, intestinal absorption, hepatic metabolism, and renal clearance [48, 50]. The findings therefore support an association between altered microbial tryptophan metabolism and diabetic bone loss, not a definitive strain‐to‐metabolite causal pathway. Defined bacterial colonization, microbial gene‐function analyses, and fecal microbiota transplantation are needed to test whether restoring IPA‐producing microbes improves skeletal outcomes. Future studies should compare exogenous IPA administration with fecal microbiota transplantation or colonization using defined IPA‐producing strains and determine whether skeletal effects depend on intact microbial IPA‐biosynthetic capacity.

Ferroptosis has emerged as a contributor to impaired osteogenesis in metabolic bone disease [51]. In osteoporosis, aging, and diabetes, iron accumulation and lipid peroxidation impair BMSC osteogenic potential and skeletal repair [52, 53]. Ferroptosis inhibition improves osteoblast function [54] and attenuates bone loss in several disease models [10]. In our study, diabetic bone showed iron deposition, lipid peroxidation, and lower GPX4 and SLC7A11 expression. HGHF‐treated BMSCs similarly accumulated Fe2+, MDA, and lipid ROS and developed mitochondrial injury. Several findings implicate ferroptosis in the response to IPA. Transcriptomic analysis identified ferroptosis‐related pathways, and IPA reduced iron accumulation and lipid peroxidation. IPA also preserved mitochondrial ultrastructure and restored GPX4, SLC7A11, and FTH1. Fer‐1 recapitulated many of the cellular and skeletal effects of IPA [55]. This transcriptional, biochemical, ultrastructural, and pharmacological evidence supports ferroptosis attenuation as one component of IPA‐mediated osteogenic protection. Other processes may contribute, because IPA also altered TNF, NF‐κB, and broader stress‐response pathways.

Our results further implicate Nrf2 as an important link between IPA and ferroptosis resistance. Nrf2 regulates genes responsible for glutathione synthesis, lipid peroxide detoxification, iron metabolism, and redox balance [56, 57]. Under glucolipotoxic conditions, Nrf2 abundance, nuclear localization, and downstream antioxidant signaling were suppressed. IPA restored the nuclear accumulation of Nrf2 and increased NQO1, SOD1, catalase, and other antioxidant genes. ML385 substantially weakened the effects of IPA on osteogenic differentiation and ferroptosis‐related proteins. These data support an important contribution of Nrf2, although genetic perturbation is needed to establish pathway specificity. The proposed gut microbiota‐IPA‐Nrf2‐ferroptosis axis therefore includes links with different levels of evidential support. Exogenous IPA administration demonstrates that IPA is sufficient to improve the measured skeletal and cellular phenotypes under the tested conditions, but it does not establish that loss of a specific IPA‐producing bacterium caused diabetic bone loss. Likewise, the ML385 experiments support a functional contribution of Nrf2, whereas molecular docking alone does not establish direct IPA‐KEAP1 binding or direct molecular activation of Nrf2. We therefore regard this axis as a working mechanistic model whose upstream microbial and direct molecular links require further experimental validation.

Current treatments for diabetic osteoporosis primarily target bone remodeling rather than upstream metabolic disturbances [12]. Modulating gut microbiota‐derived metabolites could complement these approaches by engaging host‐microbe metabolic communication [31]. IPA improved bone microarchitecture without significantly reducing hyperglycemia, suggesting direct skeletal protection despite persistent metabolic stress. BMSCs from IPA‐treated mice also retained greater osteogenic potential ex vivo, consistent with a sustained improvement in progenitor function. IPA may therefore represent a candidate postbiotic strategy for strengthening antioxidant and ferroptosis defenses. However, the current animal data do not establish therapeutic efficacy in humans. Dose‐response relationships, pharmacokinetics, long‐term safety, tissue distribution, and interactions with existing therapies require systematic evaluation. Dietary or microbial strategies to increase endogenous IPA must also produce stable, biologically relevant circulating concentrations.

Translation of IPA as a metabolite‐based postbiotic will require pharmacokinetic and pharmacodynamic characterization, including oral bioavailability, absorption, metabolism, and elimination, tissue exposure, the relationship between administered dose and achievable circulating concentrations, and the therapeutic window. Long‐term and sex‐specific safety, interactions with standard antidiabetic and anti‐osteoporosis therapies, and efficacy in larger‐animal and human studies also require evaluation. Direct IPA administration may permit more reproducible dosing, whereas diet‐, probiotic‐, or microbiota‐based approaches may support sustained endogenous production but are likely to be influenced by engraftment, diet, and interindividual microbiome variability. Although IPA did not significantly lower blood glucose in our intervention study, indirect systemic contributions cannot be excluded. We did not comprehensively assess lipid metabolism, insulin sensitivity, or inflammatory status, and changes in these parameters could influence bone remodeling independently of, or together with, a direct action on skeletal cells. The observed skeletal benefit should therefore not yet be attributed exclusively to a bone‐cell‐autonomous mechanism.

Our study also has some limitations and shortcomings. First, the human study was cross‐sectional and included only four participants per group, so the clinical findings are exploratory. Larger prospective cohorts should test whether IPA predicts bone loss or fracture after adjustment for age, sex, glycemic control, renal function, medication use, and diet. Second, the microbial source of reduced IPA was inferred from metagenomic and correlation analyses. Isotope tracing, bacterial culture, fecal transplantation, or defined colonization are needed for direct confirmation. Third, Nrf2 involvement was assessed mainly with ML385, which may have off‐target effects. Genetic loss‐ and gain‐of‐function studies are needed to confirm pathway specificity. Fourth, the predicted IPA‐KEAP1 interaction was not experimentally validated. Fifth, only male mice were studied, and sex‐dependent responses remain unknown. Finally, IPA may affect osteoclasts, immune cells, vascular cells, and other components of the bone microenvironment. The reduction in CTX‐I suggests that effects on bone resorption warrant further investigation. Accordingly, the human observations should be considered hypothesis‐generating and should not be generalized beyond this small cohort. Dedicated studies of osteoclast differentiation, activity, and resorptive function will be required to determine whether IPA directly suppresses bone resorption.

## 4 Conclusions

This study identifies an association between reduced gut microbiota‐derived IPA and impaired osteogenesis in diabetic osteoporosis. In BMSCs, IPA restored Nrf2‐associated antioxidant signaling and attenuated ferroptosis. These findings support a working model in which IPA links diabetes‐associated microbial alterations to Nrf2‐associated ferroptosis resistance and osteogenic preservation. However, the upstream microbial and direct molecular steps remain to be established. Clinical, microbial, and genetic validation will be required before this model can be considered a therapeutic target.

## 5 Experimental Section

### 5.1 Reagents and Antibodies

Indole‐3‐propionic acid (IPA; #SJ‐MX4391) and ML385 (#SJ‐MX0153) were purchased from Sparkjade (Shandong, China). Ferrostatin‐1 (Fer‐1; #HY‐100579), streptozotocin (STZ; #HY‐13753), and dimethyl sulfoxide (DMSO) were obtained from MedChemExpress. Antibodies against GAPDH (#2118), OPN (#88742), and COL1A1 (#72026) were purchased from Cell Signaling Technology. Antibodies against ACSL4 (#ET7111‐43), SLC7A11 (#HA721868), GPX4 (#ET1706‐45), FTH1 (#ET1610‐78), Nrf2 (#ER1706‐41), SOD1 (#ET1702‐36), and SP7 (#HA722817) were purchased from HUABIO. The anti‐NQO1 antibody (#bs‐2184R) was obtained from Bioss (Woburn, MA, USA). RUNX2 (#K003506P) was obtained from Beijing Solarbio Science & Technology Co., Ltd.

### 5.2 Study Participants and Serum Collection

We conducted an exploratory cross‐sectional study at Peking University Third Hospital, involving participants with diabetic osteoporosis (DMOP) and age‐ and sex‐ matched healthy volunteers as controls (CON). The study was approved by the Ethics Committee of Peking University Third Hospital (No. M20250712) in accordance with the World Medical Association's Declaration of Helsinki. Type 2 diabetes was defined in accordance with the diagnostic criteria of the World Health Organization, while osteoporosis was determined by dual‐energy x‐ray absorptiometry using a total T‐score threshold of < −2.5. Eligible participants were required to be older than 18 years. Exclusion criteria included pregnancy or lactation; metabolic or orthopedic disorders affecting bone; osteomyelitis; current or recent fracture; bone tumors; and acute diabetic complications. Participants were also excluded for hematologic or infectious disease, malignancy, autoimmune disease, or severe gastrointestinal disease. Recent exposure to glucocorticoids, immunosuppressants, antibiotics, probiotics, or other agents affecting bone metabolism or gut microbial composition was also exclusionary. Participant demographic and clinical data are presented in Table 1.

### 5.3 Animal Model and Experimental Design

All mouse experiments were approved by the Institutional Animal Ethics Committee of Peking University Third Hospital. Male C57BL/6J mice, 8 weeks of age, were obtained from the Animal Experiment Center of Peking University School of Medicine. After a 1‐week acclimatization period, the animals were randomly assigned to the designated experimental groups. To establish the diabetic osteoporosis model, mice were fed a high‐fat diet (Research Diets) for 8 weeks, followed by intraperitoneal injection of freshly prepared STZ (25 mg/kg) once daily for 5 consecutive days. STZ was prepared immediately before administration in citrate buffer (pH 4.5). Control animals received standard chow and a corresponding volume of citrate buffer. The respective diets were continued for an additional 8 weeks. After model establishment, diabetic mice were randomized to the indicated intervention groups. IPA was administered by oral gavage at 40 mg/kg five times per week, whereas Fer‐1 was delivered by intraperitoneal injection at 1 mg/kg twice weekly. Both treatments were continued for 8 weeks. IPA and Fer‐1 were dissolved in DMSO, and the control and untreated diabetic groups received equivalent volumes of DMSO by oral gavage. At the end of the intervention period, mice were euthanized by cervical dislocation. Femora and serum were subsequently collected for further analyses.

### 5.4 Serum Biochemical Analysis

Mouse serum PINP and CTX‐I concentrations were measured using ELISA kits (Elabscience Biotechnology). OCN (#WOK‐11567M1; Beijing Weioukai Technology Co., Ltd.) and IPA (#JM‐0158Z2; Jinmei Biotechnology, Inc.) were measured with the indicated ELISA kits. Human serum IPA was measured with an ELISA kit (#MM‐926075O2; Jiangsu Immunoenzyme Industry Co., Ltd.).

### 5.5 Metagenomic Sequencing and Bioinformatic Analysis

Raw metagenomic reads from mouse fecal samples were quality filtered using fastp (v0.23.4), and host‐derived reads were removed using Bowtie2 (v2.5.2). The resulting clean reads were assembled de novo using MEGAHIT (v1.2.9). Predicted genes were subsequently clustered with CD‐HIT (v4.8.1) to generate a nonredundant gene catalog, against which the clean reads were remapped using Bowtie2. Gene abundance was quantified and normalized as reads per kilobase per million mapped reads (RPKM). Taxonomic assignment was performed using Kraken2 (v2.1.3) and further refined with Bracken. Microbial diversity and downstream community analyses were performed on the Majorbio Cloud Platform.

### 5.6 Cell Isolation, Culture and Osteogenic Differentiation

Primary BMSCs were isolated from mouse femora and tibiae as previously reported [58]. Cells were maintained in alpha‐minimum essential medium (α‐MEM; Procell) supplemented 10% fetal bovine serum (FBS; #SA101.02; Cellmax). Diabetic stress was modeled in alpha‐MEM containing either 5.5 mmol/L glucose or 25.5 mmol/L glucose plus palmitic acid (300 µmol/L; MedChemExpress) [52, 59]. Osteogenesis was induced in low‐glucose DMEM (MedChemExpress) supplemented with 10% FBS and osteogenic induction supplements, as previously described [60].

### 5.7 Cell Counting Kit‐8 Assay

Cell viability was assessed at the indicated time points using a CCK8 kit (Beijing Boxbio Science & Technology Co., Ltd.) according to the manufacturer's instructions.

### 5.8 ALP and ARS Staining

At the indicated time points after osteogenic induction, BMSCs were fixed with 4% paraformaldehyde for 5 min at room temperature and sequentially rinsed with PBS and deionized water. ALP staining was performed using a commercial kit (#C3206, Beyotime), while ALP activity was measured with an alkaline phosphatase detection kit (#P0321S, Beyotime). Matrix mineralization was visualized by Alizarin Red S staining kit (#ALIR‐10001; Cyagen Biosciences, Inc.). For quantitative analysis, the retained dye was released from mineralized nodules using 10% cetylpyridinium chloride (#SJ‐MA0234, Sparkjade), and absorbance of the resulting solution was measured at 579 nm with a microplate reader (Agilent Technologies, Inc.).

### 5.9 Measurement of ROS, Mitochondrial Membrane Potential, MDA, Lipid Peroxidation, and Fe^2+^

Intracellular ROS levels were determined using a DCFH‐DA probe (MedChemExpress). Mitochondrial membrane potential was evaluated by JC‐1 staining (MedChemExpress). Cellular Fe^2^^+^ (#AKIC004M) and MDA (#AKFA013M) levels were quantified using commercial assay kits (Beijing Boxbio Science & Technology Co., Ltd.). Lipid peroxidation was assessed with the Lipid Peroxidation MDA Assay Kit (Beyotime). All assays were performed according to the manufacturers' instructions. The fluorescence signals were quantified using ImageJ (version 1.53).

### 5.10 RNA Sequencing

Raw sequencing reads were evaluated using FastQC (v0.12.1) and aligned to the mouse reference genome with STAR (v2.7.11a), using GENCODE M35 gene annotations. Transcript abundance was expressed as fragments per kilobase of transcript per million mapped reads (FPKM). Differential expression was assessed from raw gene counts using DESeq2 (v1.44.0). Genes with a fold change of ≥3 and an adjusted *p* value of <0.05 were considered differentially expressed.

### 5.11 Western Blotting Analysis

Cells were lysed in RIPA buffer (Beyotime) containing protease (#C0101) and phosphatase (#0104) inhibitors (Beijing LABLEAD Inc.), and protein concentrations were determined by BCA assay (Beyotime). Equal amounts of protein (20 µg) were loaded onto 4%–20% gradient SDS‐PAGE gels. Following electrophoresis, proteins were transferred to PVDF membranes and nonspecific sites were blocked with 5% BSA. The membranes were incubated with primary antibodies at 4°C overnight and to HRP‐linked secondary antibodies for 1 h at room temperature. After washing with TBST, chemiluminescent signals were captured with an iBright imaging system. ImageJ (version 1.53) was used for densitometric analysis, with GAPDH serving as the loading control.

### 5.12 Reverse‐Transcription Quantitative PCR

Cellular RNA was isolated with Trizol reagent and evaluated using a microvolume UV–visible spectrophotometer. Only samples with OD_260_/OD_280_ values of 1.8–2.1 were used for downstream analysis. Qualified RNA was converted to cDNA with a reverse transcription kit, followed by SYBR Green‐based amplification on a real‐time PCR system. Transcript levels were expressed relative to GAPDH and calculated using the 2^−ΔΔCt method. Table 2 lists the forward and reverse primer sequences.

**TABLE 2:** Primer sequences used for RT‐qPCR.

Gene name	Forward primer (5′‐3′)	Reverse primer (5′‐3′)
Runx2	CACCTCGAATGGCAGCACGCTA	GCCGCCAAACAGACTCATCCA
Sp7	CCTAAGGGGCACAGCTCGTCT	TGCATGTCCCACCAAGGAGTAGG
Ocn	CAGTATGGCTTGAAGACCGC	GACATCCATACTTGCAGGGC
Slc7a11	CTTTGTTGCCCTCTCCTGCTTC	CAGAGGAGTGTGCTTGTGGACA
Gpx4	GCCGAGTGTGGTTTACGAATC	GCATCGTCCCCATTTACACAG
Acsl4	AGACAAACCCGGAAGTCCAT	AGGCTGTCCTTCTTCCCAAA
Fth1	GGCTGAATGCAATGGAGTGT	TCTTGCGTAAGTTGGTCACG
Catalase	AGCGACCAGATGAAGCAGTG	TCCGCTCTCTGTCAAAGTGTG
Nqo1	AGCGTTCGGTATTACGATCC	AGTACAATCAGGGCTCTTCTCG
Sod1	AACCAGTTGTGTTGTCAGGAC	CCACCATGTTTCTTAGAGTGAGG
Gapdh	GGCAAATTCAACGGCACAGTCAAG	TCGCTCCTGGAAGATGGTGATGG

### 5.13 Molecular Docking

The three‐dimensional structure of IPA (#3744) was retrieved from PubChem, and the Kelch‐Neh2 complex structure (#2FLU) was obtained from the Protein Data Bank. Molecular docking between IPA and the Kelch‐Neh2 complex was performed using the CB‐Dock2 platform according to a previously described protocol [60].

### 5.14 Micro‐CT and Histological Analyses

Distal femora were scanned using a SkyScan micro‐CT system (Bruker Corporation), and the acquired images were reconstructed with CTvox (version 2). A region extending from 1.0 mm proximal to the distal growth plate was defined as the region of interest. Trabecular bone parameters were subsequently measured using CTAn (version 2). Following micro‐CT acquisition, femora were decalcified, embedded, and sectioned at 6 µm. HE staining was used to evaluate tissue morphology, whereas Prussian blue staining was performed to visualize iron deposition. Undecalcified specimens were subjected to von Kossa staining. Histological procedures and Immunohistochemistry were conducted according to the protocols provided by Wuhan Servicebio Technology Co., Ltd.

### 5.15 Statistical Analysis

All experimental data were presented as mean ± standard deviation (SD). Each biological experiment was performed with at least three independent biological replicates. Statistical analyses were conducted using GraphPad Prism 8.0. Comparisons between the two groups were analyzed using Student's *t*‐test. For comparisons among multiple groups (more than two groups), one‐way analysis of variance (ANOVA) followed by Tukey's posthoc test was applied. A value of *p* < 0.05 was considered statistically significant.

## Author Contributions

**Jinwu Bai**: conceptualization, methodology, data curation, visualization, Writing – original draft, project administration, investigation. **Qinyong You**: investigation, methodology, data curation, writing – original draft, visualization, project administration. **Gao Si**: investigation, data curation, software. **Daole Hu**: investigation, visualization. **Ao Sun**: investigation, visualization. **Shilong Su**: investigation, writing – original draft. **Ruideng Wang**: data curation, visualization. **Jixing Fan**: data curation, visualization. **Shan Gao**: investigation, visualization. **Tengjiao Zhu**: conceptualization, writing – review and editing, project administration, supervision. **Chun‐Li Song**: supervision, project administration, writing – review and editing, conceptualization. **Fang Zhou**: conceptualization, writing – review and editing, funding acquisition, project administration, supervision. **Yang Lv**: supervision, writing – review and editing, conceptualization, funding acquisition, project administration.

## Ethics Statement

This study was approved by the Medical Ethics Committee of Peking University Third Hospital (Approval No. M20250712). This study was conducted in strict accordance with the principles of the Declaration of Helsinki. Participants gave written informed consent.

## Conflicts of Interest

The authors declare no conflicts of interest.

## Supporting information




**Supporting File**: advs77649‐sup‐0001‐SuppMat.docx.

## Data Availability

The data that support the findings of this study are available from the corresponding author upon reasonable request.

## References

[1] Y. Dai , Y. Jiang , X. Li , et al., “Eldecalcitol Ameliorates Type 2 Diabetic Osteoporosis by Attenuating Endothelial Ferroptosis via the SOCE/O‐GlcNAcylation Axis,” Free Radical Biology and Medicine 241 (2025): 447–458, 10.1016/j.freeradbiomed.2025.09.041. 41005739

[2] L. Nagendra , K. Agarwal , S. Patra , et al., “Optimizing Bone Health in Diabetes: Strategies for Fracture Risk Reduction in Public Healthcare,” Current Diabetes Reports 25, no. 1 (2025): 43, 10.1007/s11892-025-01599-x. 40690104

[3] Z. Cai , L. Bai , Q. Li , Y. Li , X. Cai , and Y. Lin , “Gene‐Activating Framework Nucleic Acid‐Targeted Upregulating Sirtuin‐1 to Modulate Osteoimmune Microenvironment for Diabetic Osteoporosis Therapeutics,” ACS Nano 18, no. 52 (2024): 35214–35229, 10.1021/acsnano.4c08727. 39689347

[4] Y. Peng , H. Zhao , J. Chen , et al., “PGK1 Regulates Oxidative Stress in Gestational Diabetes Mellitus Through the Estradiol‐Keap1‐Nrf2 Pathway,” International Journal of Biological Sciences 21, no. 12 (2025): 5496–5513, 10.7150/ijbs.113728. 40959277 PMC12435576

[5] L. J. Raggatt and N. C. Partridge , “Cellular and Molecular Mechanisms of Bone Remodeling,” Journal of Biological Chemistry 285, no. 33 (2010): 25103–25108, 10.1074/jbc.R109.041087. 20501658 PMC2919071

[6] H. S. Shu , Y. L. Liu , X. T. Tang , et al., “Tracing the Skeletal Progenitor Transition During Postnatal Bone Formation,” Cell Stem Cell 28, no. 12 (2021): 2122–2136.e3, 10.1016/j.stem.2021.08.010. 34499868

[7] S. L. Xia , Z. Y. Ma , B. Wang , F. Gao , S. Y. Guo , and X. H. Chen , “A Gene Expression Profile for the Lower Osteogenic Potent of Bone‐Derived MSCs From Osteoporosis With T2DM and the Potential Mechanism,” Journal of Orthopaedic Surgery and Research 17, no. 1 (2022): 402, 10.1186/s13018-022-03291-2. 36050744 PMC9438120

[8] L. C. Hofbauer , B. Busse , R. Eastell , et al., “Bone Fragility in Diabetes: Novel Concepts and Clinical Implications,” The Lancet Diabetes & Endocrinology 10, no. 3 (2022): 207–220, 10.1016/S2213-8587(21)00347-8. 35101185

[9] Y.‐B. Wang , Z.‐P. Li , P. Wang , et al., “Iron Dysregulation, Ferroptosis, and Oxidative Stress in Diabetic Osteoporosis: Mechanisms, Bone Metabolism Disruption, and Therapeutic Strategies,” World Journal of Diabetes 16, no. 6 (2025): 106720, 10.4239/wjd.v16.i6.106720. 40548284 PMC12179906

[10] Z. Jiang , G. Qi , X. He , et al., “Ferroptosis in Osteocytes as a Target for Protection against Postmenopausal Osteoporosis,” Advanced Science 11, no. 12 (2024): 2307388, 10.1002/advs.202307388. 38233202 PMC10966575

[11] S. J. Dixon and J. A. Olzmann , “The Cell Biology of Ferroptosis,” Nature Reviews Molecular Cell Biology 25, no. 6 (2024): 424–442, 10.1038/s41580-024-00703-5. 38366038 PMC12187608

[12] J. Bao , Y. Yan , D. Zuo , et al., “Iron Metabolism and Ferroptosis in Diabetic Bone Loss: From Mechanism to Therapy,” Frontiers in Nutrition 10 (2023): 1178573, 10.3389/fnut.2023.1178573. 37215218 PMC10196368

[13] H.‐T. Hu , Z.‐Y. Zhang , Z.‐X. Luo , et al., “Emerging Regulated Cell Death Mechanisms in Bone Remodeling: Decoding Ferroptosis, Cuproptosis, Disulfidptosis, and PANoptosis as Therapeutic Targets for Skeletal Disorders,” Cell Death Discovery 11, no. 1 (2025): 335, 10.1038/s41420-025-02633-3. 40691135 PMC12280121

[14] F. Wei , B. Ruan , J. Dong , et al., “Asperosaponin VI Inhibition of DNMT Alleviates GPX4 Suppression‐Mediated Osteoblast Ferroptosis and Diabetic Osteoporosis,” Journal of Advanced Research 75 (2025): 331–344, 10.1016/j.jare.2024.11.036. 39647633 PMC12536592

[15] M. Yamamoto , T. W. Kensler , and H. Motohashi , “The KEAP1‐NRF2 System: A Thiol‐Based Sensor‐Effector Apparatus for Maintaining Redox Homeostasis,” Physiological Reviews 98, no. 3 (2018): 1169–1203, 10.1152/physrev.00023.2017. 29717933 PMC9762786

[16] Q. Gao , Y. Mao , S. Xie , et al., “Nrf2 Signaling in Chronic Obstructive Pulmonary Disease: Regulation of Ferroptosis and Therapeutic Implications,” Redox Biology 88 (2025): 103931, 10.1016/j.redox.2025.103931. 41252868 PMC12664812

[17] J. Wang , H. Zhuang , X. Yang , et al., “Exploring the Mechanism of Ferroptosis Induction by Sappanone A in Cancer: Insights Into the Mitochondrial Dysfunction Mediated by NRF2/xCT/GPX4 Axis,” International Journal of Biological Sciences 20, no. 13 (2024): 5145–5161, 10.7150/ijbs.96748. 39430236 PMC11488586

[18] B. Chen , Q. He , J. Yang , et al., “Metformin Suppresses Oxidative Stress Induced by High Glucose via Activation of the Nrf2/HO‐1 Signaling Pathway in Type 2 Diabetic Osteoporosis,” Life Sciences 312 (2023): 121092, 10.1016/j.lfs.2022.121092. 36279968

[19] M. M. Zaiss , R. M. Jones , G. Schett , and R. Pacifici , “The Gut‐bone Axis: How Bacterial Metabolites Bridge the Distance,” Journal of Clinical Investigation 129, no. 8 (2019): 3018–3028, 10.1172/JCI128521. 31305265 PMC6668676

[20] Z. Lyu , Y. Hu , Y. Guo , and D. Liu , “Modulation of Bone Remodeling by the Gut Microbiota: A New Therapy for Osteoporosis,” Bone Research 11, no. 1 (2023): 31, 10.1038/s41413-023-00264-x. 37296111 PMC10256815

[21] F. Indrio and A. Salatto , “Gut Microbiota‐Bone Axis,” Annals of Nutrition and Metabolism 81, no. 1 (2025): 47–56, 10.1159/000541999. 39848230

[22] H. Xu , Y. Luo , Y. An , and X. Wu , “The Mechanism of Action of Indole‐3‐Propionic Acid on Bone Metabolism,” Food & Function 16, no. 2 (2025): 406–421, 10.1039/d4fo03783a. 39764708

[23] J. Bai , G. Si , R. Wang , et al., “Gut Metabolite Indoleacrylic Acid Suppresses Osteoclast Formation by AHR mediated NF‐κB Signaling Pathway,” International Journal of Biological Sciences 22, no. 2 (2026): 951–969, 10.7150/ijbs.124766. 41522356 PMC12781171

[24] X. Xie , H. Liu , K. Wan , J. Li , and P. Qi , “The Gut Microbiota in Osteoporosis: Dual Roles and Therapeutic Prospects,” Frontiers in Immunology 16 (2025): 1617459, 10.3389/fimmu.2025.1617459. 40959063 PMC12434086

[25] Y. Hu , C. Lin , L. Zhang , X. Jiang , H. Li , and Y. Wu , “Gut Microbiota, Immunity, and Metabolism in the Progression From Chronic Liver Disease to Hepatocellular Carcinoma,” Advanced Science 13, no. 44 (2026): 23582, 10.1002/advs.202523582. PMC13353184 42429613

[26] H. Miao , S. J. Zhang , X. Wu , P. Li , and Y. Y. Zhao , “Tryptophan Metabolism as a Target in Gut Microbiota, Ageing and Kidney Disease,” International Journal of Biological Sciences 21, no. 10 (2025): 4374–4387, 10.7150/ijbs.115359. 40765836 PMC12320236

[27] S. E. Owumi , E. S. Najophe , and M. T. Otunla , “3‐Indolepropionic Acid Prevented Chlorpyrifos‐induced Hepatorenal Toxicities in Rats by Improving Anti‐Inflammatory, Antioxidant, and Pro‐Apoptotic Responses and Abating DNA Damage,” Environmental Science and Pollution Research 29, no. 49 (2022): 74377–74393, 10.1007/s11356-022-21075-3. 35644820

[28] T. Quan , R. Li , Y. Chen , and T. Gao , “Pineal Melatonin Improves Bisphenol A‐Induced Enterotoxicity via Tryptophan/AhR/Mitochondria Signaling in Pregnant Mice,” Science of The Total Environment 1005 (2025): 180867, 10.1016/j.scitotenv.2025.180867. 41202748

[29] D. Zhang , J. Wu , H. Feng , et al., “Gastrodin Ameliorates Ulcerative Colitis via Modulating Gut Microbial Tryptophan Metabolism and AhR/NLRP3 Pathway,” Phytomedicine 147 (2025): 157217, 10.1016/j.phymed.2025.157217. 40907403

[30] D. Dodd , M. H. Spitzer , W. Van Treuren , et al., “A Gut Bacterial Pathway Metabolizes Aromatic Amino Acids Into Nine Circulating Metabolites,” Nature 551, no. 7682 (2017): 648–652, 10.1038/nature24661. 29168502 PMC5850949

[31] M. Afzaal , F. Saeed , Y. A. Shah , et al., “Human Gut Microbiota in Health and Disease: Unveiling the Relationship,” Frontiers in Microbiology 13 (2022): 999001, 10.3389/fmicb.2022.999001. 36225386 PMC9549250

[32] Y.‐C. Wang , Y. C. Koay , C. Pan , et al., “Indole‐3‐Propionic Acid Protects against Heart Failure With Preserved Ejection Fraction,” Circulation Research 134, no. 4 (2024): 371–389, 10.1161/CIRCRESAHA.123.322381. 38264909 PMC10923103

[33] R. Sehgal , V. D. de Mello , V. Männistö , et al., “Indolepropionic Acid, a Gut Bacteria‐Produced Tryptophan Metabolite and the Risk of Type 2 Diabetes and Non‐Alcoholic Fatty Liver Disease,” Nutrients 14, no. 21 (2022): 4695, 10.3390/nu14214695. 36364957 PMC9653718

[34] B. Tian , H. Wang , Y. Zhang , et al., “Tryptophan‐producing Bacteria to Mitigate Osteoporosis and Intestinal Dysfunction,” Bioactive Materials 51 (2025): 293–305, 10.1016/j.bioactmat.2025.05.013. 40487243 PMC12145525

[35] C. Chen , Z. Cao , H. Lei , et al., “Microbial Tryptophan Metabolites Ameliorate Ovariectomy‐Induced Bone Loss by Repairing Intestinal AhR‐Mediated Gut‐Bone Signaling Pathway,” Advanced Science 11, no. 36 (2024): 2404545, 10.1002/advs.202404545. 39041942 PMC11423200

[36] A. Rodriguez‐Bryant , M. Papageorgiou , M. N. Horcajada , E. Biver , S. Ferrari , and N. Bonnet , “The Gut–Bone Axis: Microbial Metabolism and Nutritional Interventions for Bone Health,” Gut Microbes 18, no. 1 (2026): 2675093, 10.1080/19490976.2026.2675093. 42171633 PMC13203023

[37] T. Jiang , C. Li , Z. Pan , et al., “Gut Microbiota–Decanoic Acid–Interleukin‐17A Axis Orchestrates Hyperglycemia‐Induced Osteoporosis in Male Mice,” Diabetes 75, no. 1 (2026): 154–165, 10.2337/db25-0471. 41144501

[38] N. Chen , M. Zhang , B. Shi , et al., “Tirzepatide, a Dual GLP‐1 and GIP Receptor Agonist, Promotes Bone Loss in Obese Mice via Gut Microbial‐Related Metabolites,” Journal of Orthopaedic Translation 55 (2025): 280–292, 10.1016/j.jot.2025.09.002. 41089557 PMC12516549

[39] Y. W. Zhang , P. R. Song , S. C. Wang , H. Liu , Z. M. Shi , and J. C. Su , “Diets Intervene Osteoporosis via Gut‐Bone Axis,” Gut Microbes 16, no. 1 (2024): 2295432, 10.1080/19490976.2023.2295432. 38174650 PMC10773645

[40] Z. Li , Q. Wang , X. Huang , Y. Wu , and D. Shan , “Microbiome's Role in Musculoskeletal Health Through the Gut‐Bone Axis Insights,” Gut Microbes 16, no. 1 (2024): 2410478, 10.1080/19490976.2024.2410478. 39387683 PMC11469435

[41] H. M. Roager and T. R. Licht , “Microbial Tryptophan Catabolites in Health and Disease,” Nature Communications 9, no. 1 (2018): 3294, 10.1038/s41467-018-05470-4. PMC6098093 30120222

[42] B. Niu , T. Pan , Y. Xiao , et al., “The Therapeutic Potential of Dietary Intervention: Based on the Mechanism of a Tryptophan Derivative‐Indole Propionic Acid on Metabolic Disorders,” Critical Reviews in Food Science and Nutrition 65, no. 9 (2025): 1729–1748, 10.1080/10408398.2023.2299744. 38189263

[43] Y. Fu , J. Lyu , and S. Wang , “The Role of Intestinal Microbes on Intestinal Barrier Function and Host Immunity From a Metabolite Perspective,” Frontiers in Immunology 14 (2023): 1277102, 10.3389/fimmu.2023.1277102. 37876938 PMC10591221

[44] H. Xue , X. Chen , C. Yu , et al., “Gut Microbially Produced Indole‐3‐Propionic Acid Inhibits Atherosclerosis by Promoting Reverse Cholesterol Transport and Its Deficiency Is Causally Related to Atherosclerotic Cardiovascular Disease,” Circulation Research 131, no. 5 (2022): 404–420, 10.1161/circresaha.122.321253. 35893593

[45] R. Huang , Z.‐Y. Shen , D. Huang , et al., “Microbiota‐Indole‐3‐Propionic Acid‐Heart Axis Mediates the Protection of Leflunomide Against αPD1‐Induced Cardiotoxicity in Mice,” Nature Communications 16, no. 1 (2025): 2651, 10.1038/s41467-025-58107-8. PMC11923180 40108157

[46] H. D. Rosas , G. Doros , S. Bhasin , et al., “A Systems‐Level “Misunderstanding”: The Plasma Metabolome in Huntington's Disease,” Annals of Clinical and Translational Neurology 2, no. 7 (2015): 756–768, 10.1002/acn3.214. 26273688 PMC4531058

[47] M. Owe‐Larsson , D. Drobek , P. Iwaniak , R. Kloc , E. M. Urbanska , and M. Chwil , “Microbiota‐Derived Tryptophan Metabolite Indole‐3‐Propionic Acid‐Emerging Role in Neuroprotection,” Molecules 30, no. 17 (2025): 3628, 10.3390/molecules30173628. 40942152 PMC12429930

[48] R. Wu , Y. Kong , J. Li , et al., “Indole‐3 Propionate Inhibits NF‐κB/NLRP3‐Mediated Osteoclastogenesis and Improves Bone Quality in High‐Fat‐Diet Induced Obese Mice,” Biochimica et Biophysica Acta (BBA)—Molecular Basis of Disease 1871, no. 7 (2025): 167952, 10.1016/j.bbadis.2025.167952. 40532744

[49] R. Peng , C. Song , S. Gou , et al., “Gut Clostridium Sporogenes‐Derived Indole Propionic Acid Suppresses Osteoclast Formation by Activating Pregnane X Receptor,” Pharmacological Research 202 (2024): 107121, 10.1016/j.phrs.2024.107121. 38431091

[50] Y. Zeng , M. Guo , Q. Wu , et al., “Gut Microbiota‐Derived Indole‐3‐Propionic Acid Alleviates Diabetic Kidney Disease Through Its Mitochondrial Protective Effect via Reducing Ubiquitination Mediated‐Degradation of SIRT1,” Journal of Advanced Research 73 (2025): 607–630, 10.1016/j.jare.2024.08.018. 39147198 PMC12225927

[51] Y. Shuai , B. Chen , T. Jiang , et al., “Targeting Ferroptosis to Rescue Osteogenic Differentiation in BRONJ‐Affected Jawbone Mesenchymal Stem Cells: The Role of miR‐145‐3p and Exosome‐Mediated Therapy,” Journal of Nanobiotechnology 23, no. 1 (2025): 653, 10.1186/s12951-025-03745-9. 41074167 PMC12514850

[52] Y. Chen , W. Zhao , A. Hu , et al., “Type 2 Diabetic Mellitus Related Osteoporosis: Focusing on Ferroptosis,” Journal of Translational Medicine 22, no. 1 (2024): 409, 10.1186/s12967-024-05191-x. 38693581 PMC11064363

[53] Y. Pan , L. Chen , Y. Chen , et al., “Mitochondrial Dysfunction in Diabetic Ulcers: Pathophysiological Mechanisms and Targeted Therapeutic Strategies,” Frontiers in Cell and Developmental Biology 13 (2025): 1625474, 10.3389/fcell.2025.1625474. 40917755 PMC12408512

[54] B. Ruan , J. Dong , F. Wei , et al., “DNMT Aberration‐Incurred GPX4 Suppression Prompts Osteoblast Ferroptosis and Osteoporosis,” Bone Research 12, no. 1 (2024): 68, 10.1038/s41413-024-00365-1. 39617773 PMC11609303

[55] S. Li , Z. He , R. Zhu , et al., “An Activatable Polymeric Nano‐Spy for Reversing the Progression of Diabetic Osteoporosis,” Biomaterials 325 (2026): 123637, 10.1016/j.biomaterials.2025.123637. 40819600

[56] W. Nan , W.‐M. Zhou , J.‐L. Zi , et al., “Ferroptosis and Bone Metabolic Diseases: The Dual Regulatory Role of the Nrf2/HO‐1 Signaling Axis,” Frontiers in Cell and Developmental Biology 13 (2025): 1615197, 10.3389/fcell.2025.1615197. 40977890 PMC12446312

[57] J. Bai , G. Han , J. Fan , et al., “Gut Microbial Metabolite Alleviates Osteoporosis by Attenuating AKT‐NFATc1 Signaling Pathway and ROS Production,” Free Radical Biology and Medicine 243 (2026): 351–366, 10.1016/j.freeradbiomed.2025.11.054. 41290102

[58] J. Bai , J. Fan , R. Wang , et al., “Urolithin A Supplementation Alleviates Osteogenic Disfunction and Promotes Bone Fracture Healing in Inflammatory Environments,” Food & Nutrition Research 70 (2026): 13033, 10.29219/fnr.v70.13033. PMC13224938 42232737

[59] Y. Lin , X. Shen , Y. Ke , et al., “Activation of Osteoblast Ferroptosis via the METTL3/ASK1‐p38 Signaling Pathway in High Glucose and High Fat (HGHF)‐Induced Diabetic Bone Loss,” The FASEB Journal 36, no. 3 (2022): 22147, 10.1096/fj.202101610R. 35104016

[60] J. Bai , R. Wang , J. Fan , et al., “Microbiota‑Derived Indole‑3‑Propionic Acid Reprograms Bone Marrow Stem Cell Fate via PPARγ Suppression to Rescue Osteoporosis,” International Journal of Molecular Medicine 58, no. 2 (2026): 1–15, 10.3892/ijmm.2026.5879. PMC13252943 42246175

