# Supplementary material for: Gut Microbiota‐Derived Indole‐3‐Propionic Acid Alleviates Diabetic Osteoporosis Through Nrf2‐Mediated Ferroptosis Suppression
Source: Adv Sci (Weinh). 2026 Sep 8:e77649. Online ahead of print. doi: 10.1002/advs.77649 (PMC13554390; doi:10.1002/advs.77649)
Supplement: Supplementary file 1 [file ADVS-9999-e77649-s001.docx]

**Title:** Gut Microbiota-Derived Indole-3-Propionic Acid Alleviates Diabetic Osteoporosis Through Nrf2-Mediated Ferroptosis Suppression

Jinwu Bai^1,2, #^, Qinyong You^1,2, #^, Gao Si^1,2^, Daole Hu^1,2^, Ao Sun^1,2^, Shilong Su^1,2^, Ruideng Wang^3^, Jixing Fan^1,2^, Shan Gao^1,2^, Tengjiao Zhu^1,2, *^, Chun-Li Song1,2, *, Fang Zhou^1,2, *^, Yang Lv^1,2, *^

1 Department of Orthopedics, Peking University Third Hospital, Beijing 100191, China.

2 Beijing Key Laboratory of Advanced Bioadaptable Orthopedic Implants, Peking University Third Hospital, Beijing 100191, China.

3 Department of Trauma Orthopedics, Shenzhen Second People's Hospital (The First Affiliated Hospital of Shenzhen University), Shenzhen, 518000, China.

**Supplementary information**

Fig S1 Metabolic characterization of the STZ/HFD-induced diabetic mouse model. (A) Body weight of CON and STZ&HFD mice. (B) Fasting blood glucose concentrations at the experimental endpoint. (C) Oral glucose tolerance test (OGTT) showing impaired glucose tolerance in STZ&HFD mice. (D) Random blood glucose concentrations at the experimental endpoint.

Fig S2 Diabetic mice exhibit marked alterations in gut microbial composition and community structure. (A–C) Principal component analysis (PCA), principal coordinates analysis (PCoA), and non-metric multidimensional scaling (NMDS) demonstrating distinct clustering of gut microbial communities between CON and STZ&HFD mice. (D) Gut microbiota health index between two groups. (E) Gut microbiota dysbiosis index between two groups. (F) Analysis of the relative abundance of taxa among different groups using a LEfSe cladogram.

Fig S3 Cytotoxicity evaluation of IPA and Fer-1 in BMSCs. (A) Cell viability of BMSCs treated with increasing concentrations of IPA for 1, 2 and 3 days, determined using the CCK-8 assay. (B) Cell viability of BMSCs following treatment with increasing concentrations of Fer-1. Data are presented as mean ± SD from at least three independent experiments. *p < 0.05 **p < 0.01.

Fig S4 IPA restores the expression of osteogenic genes under diabetic conditions. (A-C) Relative mRNA expression levels of Runx2, Ocn, and Sp7 in BMSCs determined by quantitative real-time PCR. Data are presented as mean ± SD from at least three independent experiments. *p < 0.05 **p < 0.01.

Fig S5 Transcriptomic characterization of IPA-mediated regulation in BMSCs under diabetic stress. (A) Heatmap showing differentially expressed genes identified by RNA sequencing in BMSCs cultured under HGHF conditions with or without IPA treatment. (B) Heatmap of representative ferroptosis-related genes, including Hmox1, Slc40a1, Slc7a11, Gclm, and Gclc, following IPA treatment.

Fig S6 Representative immunofluorescence images of FTH1 and GPX4 in BMSCs. Nuclei were counterstained with DAPI. Scale bar, 20 µm.

Fig S7 IPA regulates the transcription of ferroptosis-associated genes in diabetic BMSCs. Relative mRNA expression levels of Gpx4, Slc7a11, Acsl4 and Fth1 in BMSCs determined by quantitative real-time PCR. Data are presented as mean ± SD from at least three independent experiments. *p < 0.05 **p < 0.01.

Fig S8 Pharmacological inhibition of Nrf2 by ML385 in BMSCs. (A) WB analysis of Nrf2 protein expression in BMSCs treated with increasing concentrations of ML385. (B) Quantification of Nrf2 protein expression normalized to GAPDH. Data are presented as mean ± SD from at least three independent experiments. *p < 0.05 **p < 0.01.

Fig S9 Cytotoxicity assessment of ML385 in BMSCs. Cell viability of BMSCs following treatment with increasing concentrations of ML385 for 1 and 3 days, determined using the CCK-8 assay.

Fig S10 Pharmacological inhibition of Nrf2 abolishes the pro-osteogenic effects of IPA under the diabetic microenvironment. (A) Representative ARS staining images of BMSCs cultured under HGHF conditions in the presence or absence of IPA and ML385. Scale bar, 25 µm. (B) Quantitative analysis of matrix mineralization determined by ARS staining. (C) Representative ALP staining images following the indicated treatments. Scale bar, 25 µm. (D) Quantification of ALP staining intensity. Data are presented as mean ± SD from at least three independent experiments. *p < 0.05 **p < 0.01.

Fig S11 ML385 reverses the inhibitory effects of IPA on ferroptosis under the diabetic microenvironment. (A) WB analysis of ferroptosis-related proteins including GPX4, SLC7A11, and ACSL4 in BMSCs following the indicated treatments. (B) Quantification of protein expression normalized to GAPDH. Data are presented as mean ± SD from at least three independent experiments. *p < 0.05 **p < 0.01.

Fig S12 Metabolic parameters following IPA and Fer-1 intervention in diabetic mice. (A) Body weight of CON, STZ&HFD, STZ&HFD+IPA and STZ&HFD+Fer-1 mice. (B) Fasting blood glucose concentrations at the experimental endpoint. (C) Random blood glucose concentrations at the experimental endpoint. n=6. Data are presented as mean ± SD. *p < 0.05 **p < 0.01.

Fig S13 (A) Serum CTX-I concentrations of the four groups. (B) Quantitative analysis of bone volume fraction based on Von Kossa staining. n=6. Data are presented as mean ± SD. *p < 0.05 **p < 0.01.

Fig S14 (A) Quantitative analysis of ARS staining following osteogenic induction of primary BMSCs isolated from four groups mice. (B) Quantitative analysis of ALP staining. Data are presented as mean ± SD from at least three independent experiments. *p < 0.05 **p < 0.01.

Fig S15 Representative H&E staining of the heart, liver, spleen, lung, and kidney collected from four groups mice. Scale bar: 100μm.
